# IoT‐Based Hand Hygiene Compliance Monitoring System and Validation of Its Effectiveness in Hospital Environments

**DOI:** 10.1002/gch2.202400124

**Published:** 2024-11-12

**Authors:** Ju‐Yu Wu, Yi‐Chun Lin, Shu‐Yuan Lee, Cheng‐Pin Chen, Shu‐Hsing Cheng, Chien‐Yu Cheng, Congo Tak Shing Ching, Hui‐Min David Wang, Chu‐Chun Yeh, Wei J. Chen, Wei‐Wen Chen, Lun‐De Liao

**Affiliations:** ^1^ Institute of Biomedical Engineering and Nanomedicine National Health Research Institutes 35, Keyan Road, Zhunan Town Miaoli County 350 Taiwan; ^2^ Doctoral Program in Tissue Engineering and Regenerative Medicine National Chung Hsing University 145, Xingda Road, South District Taichung City 402 Taiwan; ^3^ Department of Infectious Diseases Taoyuan General Hospital Ministry of Health and Welfare Taoyuan City 330 Taiwan; ^4^ Graduate Institute of Clinical Medicine, College of Medicine Taipei Medical University Taipei 110 Taiwan; ^5^ School of Clinical Medicine National Yang Ming Chiao Tung University Taipei City 112 Taiwan; ^6^ School of Public Health Taipei Medical University Taipei City 110 Taiwan; ^7^ Institute of Public Health School of Medicine National Yang‐Ming Chiao Tung University Taipei City 112 Taiwan; ^8^ Graduate Institute of Biomedical Engineering National Chung Hsing University 145, Xingda Road, South District Taichung City 402 Taiwan; ^9^ Department of Electrical Engineering National Chi Nan University 1 University Road, Puli Township Nantou County 545301 Taiwan; ^10^ International Doctoral Program in Agriculture National Chung Hsing University 145, Xingda Road, South District Taichung City 402 Taiwan; ^11^ Advanced Plant and Food Crop Biotechnology Center National Chung Hsing University 145, Xingda Road, South District Taichung City 402 Taiwan; ^12^ Department of Infectious Control Taoyuan General Hospital Ministry of Health and Welfare Taoyuan City 330 Taiwan; ^13^ Department of Nursing Taoyuan General Hospital Ministry of Health and Welfare Taoyuan City 330 Taiwan; ^14^ Center for Neuropsychiatric Research National Health Research Institutes 35, Keyan Road, Zhunan Town Miaoli County 350 Taiwan; ^15^ Institute of Biomedical Engineering National Yang Ming Chiao Tung University Hsinchu City 300093 Taiwan

**Keywords:** active handwashing observation system, hand hygiene, healthcare, intelligent systems, IoT, public health

## Abstract

Healthcare‐associated infection (HAI) is the most common adverse medical event that affects patients. Internationally, healthcare workers (HCWs) are monitored for hand hygiene compliance to reduce HAI risk. While direct observation is considered the gold standard for monitoring, it has several disadvantages. To address this, the study focused on developing a comprehensive hand hygiene system that integrates the Internet of Things (IoT) hand hygiene with soap and water (HHW) and alcohol‐based formulation (HHA) monitoring, incorporates real‐time data visualization on a web interface to track HCWs' hand hygiene practices, and provides instant calculations of compliance and accuracy rates. This IoT system uses Bluetooth for HCW positioning and HHW detection, ultrasonic sensors for handwashing duration, and pressure sensors for HHA detection. Furthermore, a cloud server, database, and website are established to manage and display the data received by the IoT devices. To reduce HAI in Taiwan, hospitals must provide both HHW and HHA systems, and HCWs can choose either method when hand hygiene is necessary. The system achieved 72% accuracy in clinical practice within an adult intensive care unit (ICU).

## Graphical abstract

The article presents an advanced IoT‐based hand hygiene compliance monitoring system designed to enhance healthcare‐associated infection (HAI) prevention. Utilizing Bluetooth Low Energy (BLE) for precise location tracking and ultrasonic sensors for handwashing duration detection, the system achieves high accuracy in controlled environments. Integrated with a cloud server and real‐time data visualization via Streamlit, it offers an automated solution for monitoring hand hygiene compliance, overcoming the limitations of traditional observation methods. Further research is required to optimize its effectiveness in clinical settings and reduce HAIs.

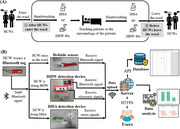

## 1 Introduction

The World Health Organization (WHO) Guidelines on Hand Hygiene in Health Care state that healthcare‐associated infection (HAI or HCAI) affects hundreds of millions of patients annually. HAI is the most common medical condition affecting patients and is the root cause of many adverse events.^[^ ^1^, ^2^, ^3^, ^4^, ^5^ ^]^ HAI can have many undesirable effects, worsening the condition of patients and thus prolonging the hospitalization period, increasing the disability and mortality rates among patients, and increasing the economic burden on personnel and the healthcare system.^[^ ^6^ ^]^ In addition, HAI is very common in the intensive care unit (ICU). According to a prevalence study conducted in Europe, ≈19.2% of patients in the ICU experienced at least one HAI.^[^ ^7^ ^]^

The risk of HAI is high and is caused by many factors related to the nursing system and poor hand hygiene habits.^[^ ^1^ ^]^ Thus, ≈20% to 70% of HAIs are preventable. Unsuitable hand hygiene habits can result in the spread of various bacteria, especially in medical institutions.^[^ ^8^, ^9^ ^]^ Proper hand hygiene is critical for preventing cross‐transmission of HAIs and multidrug‐resistant microorganisms.^[^ ^10^, ^11^, ^12^ ^]^ However, the median hand hygiene compliance rate in medical institutions worldwide is only 40%.^[^ ^13^, ^14^ ^]^ Research shows that many elements can lead to poor hand hygiene habits, such as the personal and professional characteristics of healthcare workers (HCWs); hospital ward types; workload; the temperature of the tap water; the availability of soap, hand sanitizer, and paper towels; the urgency of emergency interventions; understaffing; overcrowding; and administrative penalties and incentives.^[^ ^6^, ^10^, ^15^ ^]^

Interventions such as education, motivation, regular direct or indirect monitoring and feedback, and behavior management can help solve this problem.^[^ ^16^, ^17^ ^]^ However, behavioral scientists emphasize that the promotion of handwashing should avoid damaging self‐efficacy through a message of fear or disgust. Once the motivation to wash hands is aroused, interventions in the form of action plans and reminders can help transform the intentions of individuals into actions.^[^ ^17^ ^]^ In addition, the WHO recommends the use of a “multimodal hand hygiene improvement strategy” and the “Five Moments for Hand Hygiene” to promote hand hygiene. The multimodal hand hygiene improvement strategy includes the provision of alcohol‐based hand sanitizers at the point of care, education and review of hand hygiene behaviors for HCWs’ performance feedback, workplace reminders, and an institutional safety culture.^[^ ^10^, ^11^ ^]^ The Five Moments for Hand Hygiene emphasize that hand hygiene behaviors should be performed before contact with the patient, before performing clean/sterile operations, after contact with the patient's body fluids, after contact with the patient, and after contact with the patient's surrounding environment (Figure S1A, Supporting Information). Performing hand hygiene at these five time points can help reduce the spread and transmission of germs and cross‐infections.^[^ ^18^ ^]^

There are many ways to monitor hand hygiene compliance in medical and healthcare institutions. Hand hygiene compliance refers to following a series of established operating procedures to ensure that hand hygiene is effectively performed. The compliance rate can be evaluated on the basis of direct observation, nurse diaries, or records from body‐worn manual counting devices.^[^ ^19^, ^20^ ^]^ Direct observation is considered the gold standard for monitoring hand hygiene; it is usually performed by trained observers^[^ ^21^, ^22^, ^23^, ^24^ ^]^ and provides immediate feedback.^[^ ^1^, ^6^, ^10^, ^11^, ^16^, ^17^, ^19^, ^21^, ^22^ ^]^ However, owing to human resource limitations, subjectivity, expense, discontinuity, time and resource consumption, insufficient sample size, a lack of standardization of observation practices, and the Hawthorne effect, such observers can record only < 1%–2% of hand hygiene events.^[^ ^19^ ^]^

Because of these limitations, there is no ideal method for monitoring hand hygiene or collecting related data. Thus, the development of relative electronic monitoring systems has evolved rapidly in recent years.^[^ ^15^, ^25^ ^]^ Various technologies for wireless networking and the Internet of Things (IoT) are utilized in medical environments as alternative or complementary monitoring methods^[^ ^12^, ^23^ ^]^ for automatic handwashing activity detection.^[^ ^26^ ^]^ On this basis, in this research, a handwashing detection system based on IoT technology was developed that uses Bluetooth to determine whether a medical staff member has entered the ward and estimate the duration of their presence. Moreover, Bluetooth and ultrasonic sensors are used to sense whether a specific HCW performs hand hygiene with soap and water (HHW) and disinfection duration. A pressure sensor is further used to detect the pressure and duration of hand hygiene with an alcohol‐based formulation (HHA).^[^ ^27^ ^]^ The proposed system can overcome the difficulties of direct observation, minimize the impact on the medical process of medical staff, and avoid the privacy issues presented by cameras.^[^ ^28^, ^29^ ^]^ Furthermore, the developed system can actively monitor the hand hygiene behaviors of HCWs and supplement direct observation by trained observers.

## 2 Experimental Section

### 2.1 Experimental Design

Design science research methodology (DSRM) was applied in this study to observe and analyze the current situation, discover solutions, and design related sensors. Following preliminary experiments, simulations and field studies were conducted to evaluate, discuss, and adjust the performance of the sensors in the field. In this study, a system was designed with the goal of avoiding interference with the workflow routines of medical staff while automatically monitoring their handwashing behaviors. Therefore, radio frequency identification (RFID) technology based on passive swipe cards or worn bracelets could interfere with medical staff workflow routines. Camera monitoring technology might record the faces of people in the ICU, which presents privacy issues. As a result, both the RFID and the camera were excluded. Instead, Bluetooth, ultrasonic, and pressure detection technologies were chosen for automatic monitoring.^[^ ^30^, ^31^ ^]^

**Figure 1:**
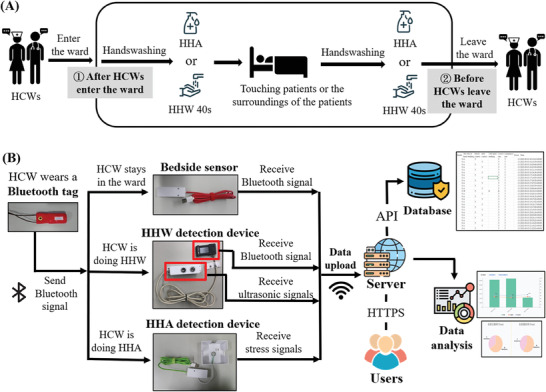
Introduction to handwashing procedures and data transmission. A) The handwashing procedures used in the present study. When an HCW remains in a ward longer than 3 min, an HHW action should last more than 40 s. B) The simplified data transmission process. The Bluetooth tags send Bluetooth signals that are received by the bedside sensor and the HHW detection device. The sensors transmit data to the server to be stored in the database for data analysis and displayed on the website.

According to the Five Moments for Hand Hygiene identified by the WHO and the hand hygiene standards and nursing practices at Taoyuan Hospital of the Ministry of Health and Welfare, the occasions for hand hygiene and the associated behaviors are clearly stipulated. Handwashing behavior must comply with the hand hygiene instructions in the WHO's Guidelines on Hand Hygiene in Health Care (Figure S1B and C, Supporting Information). However, because of the limited deployment of IoT sensor technology, the behaviors of HCWs cannot be detected after they enter the ward. Therefore, a set of handwashing rules was established in this research. HHW or HHA should be completed after entering and before leaving when the HCW remains in the ward for longer than 3 min. Moreover, an HHW action should last more than 40 s, and an HHA action should be counted only once within a duration of 5 s to prevent the data from being misrecorded and misrecognized (**Figure** **1**A).

At the experimental site where this study was performed, the adult ICU had six wards without doors and two wards with doors. In accordance with the hand hygiene regulations of the Taiwan Centers for Disease Control, every intensive care unit should be equipped with both a hand sanitizing system and a hand washing system.^[^ ^32^ ^]^ Therefore, HCWs could use alternative HH methods to clean their hands. The HCWs responsible for these eight wards must be equipped with Bluetooth tags. In addition, one bedside sensor, an HHW detection device, and an HHA detection device were deployed in every ward. Each device has a unique MAC address, which can be used to distinguish specific workers and wards. A Bluetooth signal was continuously transmitted through each Bluetooth tag. The bedside sensor was installed on the wall at the head of the hospital bed and detects Bluetooth signals every 30 s to determine how long an HCW had been present in the ward. For HHW detection, a Bluetooth signal receiver was used in combination with an ultrasonic sensor directly at the back of the faucet and sink. The Bluetooth signal receiver detects the strongest Bluetooth signal to identify the nearest HCW to the HHW station to confirm which HCW was washing their hands. The ultrasonic sensor could detect objects within 10 cm of the sink to determine how long the HCW had been performing hand hygiene. For HHA detection, a pressure sensor was placed under the bottle of alcohol‐based hand sanitizer to detect whether the user had performed an HHA action.

The signals were detected and uploaded to a server through Wi‐Fi. Additionally, the uploaded signals were stored in a cloud database, and a program was run to eliminate noise, which was affected by decorations and other mechanical noise in the clinical environment. The data were then sorted. The data were subsequently analyzed to calculate the device accuracy rate and the hand hygiene compliance and accuracy rates. The relevant formulas ((1), (2), (3), (4), (5)) of the device accuracy rate were as follows:

(1) $$\mathrm{Accuracy}\;\mathrm{of}\;\mathrm{the}\;\mathrm{whole}\;\mathrm{system}\;=\frac{\mathrm{Number}\;\mathrm{of}\;\mathrm{correct}\;\mathrm{detections}\;\mathrm{by}\;\mathrm{the}\;\mathrm{system}}{\mathrm{Total}\;\mathrm{number}\;\mathrm{of}\;\mathrm{field}\;\mathrm{inspection}\;\mathrm{data}\;\mathrm{entries}}\;\times 100\%$$

(2) $$\mathrm{Accuracy}\;\mathrm{of}\;\mathrm{the}\;\mathrm{bedside}\;\mathrm{sensor}\;=\frac{\mathrm{Number}\;\mathrm{of}\;\mathrm{incidents}\;\mathrm{in}\;\mathrm{which}\;\mathrm{the}\;\mathrm{device}\;\mathrm{correctly}\;\mathrm{detected}\;\mathrm{people}\;\mathrm{in}\;\mathrm{the}\;\mathrm{ward}}{\mathrm{Number}\;\mathrm{of}\;\mathrm{events}\;\mathrm{involving}\;\mathrm{people}\;\mathrm{in}\;\mathrm{the}\;\mathrm{ward}\;\mathrm{according}\;\mathrm{to}\;\mathrm{the}\;\mathrm{field}\;\mathrm{inspection}}\;\times 100\%$$

(3) $$\mathrm{Accuracy}\;\mathrm{of}\;\mathrm{the}\;\mathrm{HHA}\;\mathrm{detection}\;\mathrm{device}\;=\frac{\mathrm{Number}\;\mathrm{of}\;\mathrm{HHA}\;\mathrm{actions}\;\mathrm{correctly}\;\mathrm{detected}\;\mathrm{by}\;\mathrm{the}\;\mathrm{device}}{\mathrm{Number}\;\mathrm{of}\;\mathrm{HHA}\;\mathrm{actions}\;\mathrm{according}\;\mathrm{to}\;\mathrm{the}\;\mathrm{field}\;\mathrm{inspection}}\;\times 100\%$$

(4) $$\mathrm{Accuracy}\;\mathrm{of}\;\mathrm{the}\;\mathrm{HHW}\;\mathrm{detection}\;\mathrm{device}\;=\frac{\mathrm{Number}\;\mathrm{of}\;\mathrm{HHW}\;\mathrm{actions}\;\mathrm{correctly}\;\mathrm{detected}\;\mathrm{by}\;\mathrm{the}\;\mathrm{device}}{\mathrm{Number}\;\mathrm{of}\;\mathrm{HHW}\;\mathrm{actions}\;\mathrm{according}\;\mathrm{to}\;\mathrm{the}\;\mathrm{field}\;\mathrm{inspection}}\;\times 100\%$$

(5) $$\mathrm{Noise}\;\mathrm{rate}\;=\frac{\mathrm{Number}\;\mathrm{of}\;\mathrm{detection}\;\mathrm{not}\;\mathrm{corresponding}\;\mathrm{to}\;\mathrm{correct}\;\mathrm{events}}{\mathrm{Total}\;\mathrm{number}\;\mathrm{of}\;\mathrm{field}\;\mathrm{inspection}\;\mathrm{data}\;\mathrm{entries}}\;\times 100\%$$

The formulas for the compliance and accuracy rates of hand hygiene are as follows:

(6) $$\mathrm{Compliance}\;\mathrm{rate}\;\mathrm{of}\;\mathrm{hand}\;\mathrm{hygiene}\;=\frac{\mathrm{Actual}\;\mathrm{number}\;\mathrm{of}\;\mathrm{hand}\;\mathrm{hygiene}\;\mathrm{actions}\;\mathrm{performed}\;\mathrm{when}\;\mathrm{they}\;\mathrm{should}\;\mathrm{be}}{\mathrm{Total}\;\mathrm{number}\;\mathrm{of}\;\mathrm{hand}\;\mathrm{hygiene}\;\mathrm{actions}\;\mathrm{that}\;\mathrm{should}\;\mathrm{be}\;\mathrm{performed}}\;\times 100\%$$

(7) $$\mathrm{Accuracy}\;\mathrm{rate}\;\mathrm{of}\;\mathrm{hand}\;\mathrm{hygiene}\;=\frac{\mathrm{Number}\;\mathrm{of}\;\mathrm{correct}\;\mathrm{hand}\;\mathrm{hygiene}\;\mathrm{actions}\;\mathrm{performed}\;\mathrm{when}\;\mathrm{they}\;\mathrm{should}\;\mathrm{be}}{\mathrm{Total}\;\mathrm{number}\;\mathrm{of}\;\mathrm{hand}\;\mathrm{hygiene}\;\mathrm{actions}\;\mathrm{that}\;\mathrm{should}\;\mathrm{be}\;\mathrm{performed}}\;\times 100\%$$

Finally, the actual hand hygiene status was displayed on a webpage, which administrators could periodically check (Figure 1B). According to the experimental design, at least two hand‐cleaning records, such as two HHWs, two HHAs, or one HHW plus one HHA, should be detected during the time when an HCW was present in a ward. The compliance rate represents whether HCWs clean their hands at the right time. In contrast, the accuracy rate reflects whether HCWs wash their hands at the right time and for the correct duration. These indicators could be used to assess the hand hygiene behavior of HCWs when they enter and exit wards, which was part of the WHO hand hygiene guidelines. However, the system cannot fully monitor the five moments of hand hygiene guidelines proposed by the WHO because the current IoT technology cannot follow vague principles, such as no actual time and no actual distance, and turn it into a program.

### 2.2 Sensors

The hand hygiene monitoring system includes four IoT devices: a bedside sensor, an HHW detection device, an HHA detection device, and a Bluetooth tag. These sensors could detect the movements of HCWs and then upload data to the server to be stored in the cloud database for subsequent analysis, aggregation, management, hygiene control, and browsing via the website. The various sensor components were shown in Figure S2 (Supporting Information). Moreover, Figure S3 (Supporting Information) shows that each sensor had four essential parts: a sensing/identification unit, a processing unit, a power unit, and a communication unit. Considering the circumstances in the ICU and convenience of used, wireless technology was adopted as much as possible for data transmission.^[^ ^33^ ^]^ Thus, the communication units were based mainly on Bluetooth low energy (BLE) and Wi‐Fi technologies. All the devices were introduced in detail in the following subsections.

#### 2.2.1 Bedside Sensor

In the ideal case, the bedside sensor detects whether HCWs are near the patient or performing medical treatment. Each record was then labeled with the corresponding identity, the duration of the event, and the total length of time in the ward. Finally, the data are uploaded and stored in the cloud database through Wi‐Fi for subsequent data processing. The ESP32 DevKit, which was produced by Espressif Systems, was the core component of the bedside sensor and had BLE scanning and Wi‐Fi capabilities to meet the application requirements. BLE scanning was used to detect the presence of Bluetooth tag signals near the patient and determine the identities of the detected HCWs, and the data were then uploaded to the cloud database through Wi‐Fi.

#### 2.2.2 HHW Detection Device

The primary functions of the HHW detection device under ideal conditions were to detect the Bluetooth signal of an HCW who was near the sink to wash their hands and to sense the duration of handwashing to determine whether the handwashing time meets the minimum requirement established in the hospital. The LCD of the HHW host should display the current duration of the hand hygiene action to remind the HCW to properly complete the HHW procedure. The HHW host should also be able to receive, record and identify a Bluetooth signal. Information about each recorded event, such as the corresponding time points and handwashing duration, should be uploaded to the cloud database. Owing to the complex functions of this device, it may not be easy to use a single‐chip host. Although laptops can meet the corresponding requirements, they have certain limitations, such as high price, excessive size, and difficulty in installation and maintenance. Thus, other control chips should preferentially be chosen for the HHW host.

On the basis of the above considerations, the LILYGO T‐PicoC3 was chosen as the main module of the system and paired with HC‐SR04 modules and a full‐color LCD to fulfill the necessary functions of the HHW detection device. The LILYGO T‐PicoC3 was a dual‐processor motherboard with high‐efficiency processing capabilities, low power consumption, and Wi‐Fi and Bluetooth connectivity functions. It could simultaneously handle the real‐time display and update of information on the LCD screen and the sensing of the ultrasonic sensor. Moreover, it could scan and identify Bluetooth tag signals and upload related data to the cloud server through Wi‐Fi. Furthermore, the LILYGO T‐PicoC3 had a watchdog timer. When the system stops due to program execution, Watchdog could automatically reset the system within a set time, meaning that manual restart of the system was not necessary. Overall, the HHW system had a low cost, a small size, stable power consumption, and good reliability.

The HC‐SR04 ultrasonic sensor was widely used to measure distance in various IoT systems. The sensor emits ultrasonic waves and performs noncontact ranging calculations by monitoring the ultrasonic pulses and their reflection times. Because of the limitations of this sensor, fine‐grained estimation of the behaviors of HCWs was not possible. Therefore, the number of seconds for which an HCW was detected to remain within a certain distance in front of the sink was considered the handwashing time.

#### 2.2.3 HHA Detection Device

The HHA detection devices record information, such as the time and ward number, for every event in which an HWC performs HHA in a ward. This information should be uploaded to the cloud database through Wi‐Fi. Therefore, the ESP32 DevKit was again chosen as the core component, and it was combined with an FSR 402 resistive film pressure sensor and a self‐designed mechanism printed with a 3D printer to meet the requirements of the HHA detection device. Notably, many factors, such as the amount of pressure applied by the user and the remaining weight of the sanitizer spray bottle, could affect the interpretation of whether alcohol‐based sanitizer has been used and whether the HCW had performed HHA action. Therefore, a simple push‐button mechanism was not adequate. Instead, a higher‐cost membrane‐type pressure sensor was used as the main system component. The accuracy of interpretation had been improved on the basis of the results of multiple experiments and iterative program development.

#### 2.2.4 Bluetooth Tag

Bluetooth tags were used for personal identification. Therefore, battery operation, long‐term usage, small size, light weight, and reusability were desirable.^[^ ^34^ ^]^ BLE technology combined with a built‐in rechargeable lithium battery and charging circuit was essential to fulfill these needs. Although the size and weight of the chosen Bluetooth tag design were slightly inferior to those of a design based on disposable batteries, the chosen design was more appropriate from the perspective of environmental protection.

### 2.3 Sensor Evaluations

Evaluating IoT devices before introducing them into clinical applications was extremely important. In this research, separate functional tests were conducted on the bedside sensor, the HHW detection device, and the HHA detection device. The combination of all three detection devices functioning as a complete handwashing detection system was also tested, and then a simulation of an actual medical site was performed. A full sandbox test of the handwashing procedures was subsequently conducted by HCWs in an actual ward. Data were collected to assist in adjusting the parameters and algorithms. Usage habits could also serve as a reference for system fine‐tuning. Ensuring the accuracy and reliability of these devices before their clinical application is essential to support further development of the system, which could lead to its broader adoption for real‐time infection monitoring to reduce risks in medical institutions.

#### 2.3.1 Determination of the Proper Wearing Position of the Bluetooth Tag

Bluetooth is a wireless communication technology. The 2.4 GHz ISM band was used in this study. However, Bluetooth signals in this band could be easily absorbed when attempting to pass through the human body. Many factors, such as the moisture content of body tissue, the type of body tissue, and the reflection and refraction of electromagnetic waves in the tissue, could affect the distortion and attenuation of Bluetooth signals, causing interference with signal reception. To prevent errors caused by such Bluetooth distortion in the developed handwashing detection system, different packaging materials for the Bluetooth tags were tested in this research to increase the distance between the tag and the body; the tested distances were 0, 7, 12, and 20 mm. Because the Bluetooth tag should not affect the work‐related operations of the HCW wearing it, the maximum feasible distance between the Bluetooth tag and the human body was considered 20 mm. The bedside sensor was subsequently used to detect the received signal strength indicator (RSSI) intensity to determine what type of packaging material was most suitable for reducing the absorption of Bluetooth signals by the human body.

#### 2.3.2 Evaluation of the Bedside Sensor

The purpose of the bedside sensor was to detect when HCWs wearing Bluetooth tags enter the ward, when they leave the ward, and how long the HCWs were in the ward. Therefore, in this study, the distance between the Bluetooth tag and the bedside sensor was set to 0.3, 2, 4, 6, and 10 m. The bedside sensor was used to detect the RSSI intensity to determine whether HCWs were present in the ward. The duration for which the Bluetooth tags remained in the ward was also used to adjust the resolution of the bedside sensor to accommodate the actual behaviors of HCWs.

#### 2.3.3 Evaluation of the HHW Detection Device

The HHW detection device detects the handwashing time and the specific identity of the HCW performing the handwashing action. Therefore, the relationship between the position of the HHW host and the Bluetooth tag should be examined to determine the best method of wearing the Bluetooth tag and the best placement of the HHW host. There will be only one specific HCW performing HHW in a ward at a given time. However, the experimental site had many open wards, and many HCWs wearing Bluetooth tags were present onsite. Therefore, interference with the HHW detection device was examined in a simulated environment with multiple Bluetooth tags. The distances between the HHW host and the Bluetooth tags were set to 100, 150, 200, 230, 330, 340, 350, 400, 480, and more than 600 cm to evaluate the accuracy of the HHW host in detecting a specific Bluetooth tag (Figure S4A, Supporting Information). In addition, to improve the accuracy of the HHW detection device, partitions with different shapes and sizes were tested.

Moreover, many types of verified medical equipment are present in the clinical environment. Therefore, the HHW host was also tested in a multi‐interference environment (Figure S4B, Supporting Information). Considering that the wall between two adjacent wards was not thick enough to block signal interference, the interference between two HHW hosts was tested. Tests were performed at distances of 330, 450, and 950 cm between the two HHW hosts and with a metal partition placed at a distance between 450 and 950 cm (Figure S4C, Supporting Information). The accuracy of the HHW host in detecting a specific Bluetooth tag was calculated to ensure the proper functioning of the WWH detection device when used in actual clinical practice.

#### 2.3.4 Evaluation of the HHA Detection Device

Two main factors affect the accuracy of the pressure sensor in the HHA detection device: the need for HCW to obtain sufficient alcohol‐based hand sanitizer, which affects the pressing force applied by the HWC, and the amount of sanitizer remaining in the spray bottle. The new bottle contained 1000 ml of alcohol‐based hand sanitizer. To prevent the bottle content from being too low and unable to be detected correctly, spray bottles with 250 and 500 ml of sanitizer remaining were used in this study. Different shapes and lengths of the pressure plate under the spray bottle were examined to maximize the detection sensitivity of the pressure sensor. Moreover, various subjective pressing forces were tested by asking 8 users to apply light, medium, or heavy pressure to determine the threshold for dispensing sufficient sanitizer from the HHA device.

#### 2.3.5 Sandbox Test of the Handwashing Detection System

The handwashing detection system was used to analyze 16 basic handwashing behaviors performed in three different simulation environments and by different users. The first test site was an interference‐free environment in the laboratory, and in this environment, eight non‐HCWs performed the 16 handwashing behaviors (Figure S4D, Supporting Information). The second test site was in the clinical area of the hospital, and the engineers executed the 16 handwashing behaviors eight times (Figure S4E, Supporting Information). The final test was also performed in the hospital's clinical area, but eight HCWs were asked to simulate the 16 handwashing behaviors (Figure S4E, Supporting Information). The results from all three situations were recorded and uploaded to the cloud database for further qualitative and quantitative analyses to fine‐tune the actual usage of the developed system in the field. To calculate the individual device accuracy rate and the noise rate, Equations (1)–(5) were used.

#### 2.3.6 Actual Test of the Handwashing Detection System in a Clinical Environment

The handwashing detection system was deployed in a real clinical environment to compare actual HCW behaviors with the data received from all the sensors. Infection control nurses performed examinations of handwashing behaviors at three different times, spanning four hours in total. The results of this manual audit were compared with the detection results of the developed system to calculate the accuracy of the whole system, the accuracy of individual detection devices, and the noise rate to analyze the reliability and usability of this system in actual field applications. Equations (6) and (7) were used to calculate the compliance and accuracy rates of hand hygiene.

### 2.4 Architecture of the IoT Software and Access to the Cloud Database

A representational state transfer (REST) architecture, which is based on the hypertext transfer protocol (HTTP) in combination with the OpenAPI standard and Python for programming, was adopted in this study. The HTTP communication protocol was chosen for the many wireless communication devices utilized in this study because of its stateless nature. After transmission was completed, the connection was disconnected, which could lower the system operating costs and the burden on IoT devices. Therefore, embedded devices could be chosen rather than higher‐cost equipment with more robust performance.

Every detection device in every ward was equipped with Wi‐Fi communication capabilities for data collection. The IEEE 802.11 protocol was used to directly connect to the 4G network through a Wi‐Fi access point to communicate with the local network server. The local network server collects the hand hygiene events detected by all the detection devices and stores the data in a Raspberry Pi. The data could be transmitted to the cloud database through a virtual private network (VPN) established on the basis of the physical network. To limit the burden on the cloud database, there was no direct communication between different detection devices. Therefore, there was also no need to frequently open or close connections between the many detection devices and the cloud database. Only fixed links to the data collection application need to be considered (Figure S5, Supporting Information).

The timestamp of each data entry recorded in the local network was used to distinguish the sequence of actions to determine when the nursing staff entered and exited each ward and when they washed their hands. The designed architecture enables smooth and effective data collection and management, and the information security was sufficient to ensure the integrity and reliability of the data. The technology and architecture design guarantee both the efficiency and cost‐effectiveness of the system while also providing sustainability and scalability. Thus, the architecture design was critical to the performance of the system developed in this research (Figure S6, Supporting Information).

### 2.5 Clinical Data Analysis Process

There were five steps in the clinical data analysis process: data preprocessing, handwashing time estimation, handwashing data recording, data analysis, and web page deployment. First, after the raw data were downloaded, the data were sorted, the noise was deleted, and the MAC address was transformed into the corresponding ward number, detection device ID, or Bluetooth tag ID. Second, the duration of detection of each Bluetooth tag by the bedside sensor, which represents how long the corresponding HCW remained in the ward, was estimated. An event with a duration of less than 3 min was considered noise and deleted. After the time range was confirmed, handwashing records during this period could be found. If an HHW action had been detected, the bedside sensor and the HHW Bluetooth detector should simultaneously detect the signal from the same Bluetooth tag in the same room. Therefore, a record was counted only when all three sets of data were consistent. Finally, the hand hygiene compliance rates and accuracy rates were calculated and displayed on the web page after the number of handwashing actions, timing, Bluetooth tag signals, and other related information were compared (Figure S7, Supporting Information).

The meanings of the compliance rates and accuracy rates of hand hygiene differ as follows. The compliance rate of hand hygiene was calculated as the proportion of instances in which HCWs performed hand hygiene behaviors at the right time. The duration of HHW and the amount of sanitizer used in HHA were not relevant to this calculation, which means that the compliance rate does not reflect how well hand hygiene guidelines were implemented in practice. In contrast, the accuracy rate of hand hygiene was relatively strict. The correct timing of hand hygiene actions, the duration of HHW actions, and the quantity of alcohol‐based sanitizer used in HHA actions were all considered in its calculation.

### 2.6 User Interface of the Handwashing Detection System

With the rapid developments in data science and artificial intelligence that have occurred in recent years, the demand for interactive data visualizations and real‐time presentation tools had gradually increased. Therefore, dynamic and interactive display capabilities for research results have become essential in scientific and engineering practice. Accordingly, the data collected in the developed system, such as the times when HCWs enter and exit a ward, handwashing timing, etc., were rendered on a website using Streamlit. Streamlit was an open‐source Python library that could serve as a web framework owing to the provision of a series of application programming interfaces (APIs). Various interactive elements, such as charts, text outputs, and buttons, could be created in Python, and the elements instantly appear on the web page. Streamlit's internal architecture runs on the local server. When corresponding scripts were executed on the local host, the web server starts to render the data analysis, visualization, and interactive content on the web page. In this way, an interactive web page was generated that provides hand hygiene compliance and accuracy rates for a specific time. Testing, adjustments, and modifications in the local environment and the enhancement of data security and privacy could be ensured through the design pattern.

**Figure 2:**
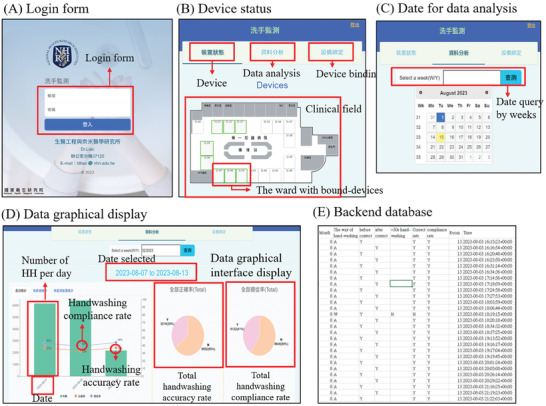
The user interface of the handwashing detection system. A) Login page. B) Device status. After a user logs in and their usage permissions are confirmed, the device status, data analysis, and device binding settings are displayed. C) The selection of dates for data analysis. D) Graphical display of the data. The accuracy and compliance rates are shown in the graphics after the relevant data are collected and processed. E) Backend database.

After a user logs in on the website and their usage permissions are confirmed (**Figure** **2**A), the device status, data analysis, and device binding status were displayed (Figure 2B). The specific week could be chosen (Figure 2C), and the number of handwashing events, the accuracy rate, and the compliance rate were displayed in a graphical interface after the relevant data were collected and processed (Figure 2D). The daily compliance rates and accuracy rates of hand hygiene behaviors over one week were displayed in a line chart. This allows health authorities to easily perform daily data comparisons when conducting intervention research on handwashing strategies. In addition, the weekly hand hygiene accuracy and compliance rates were displayed in pie charts, where the percentages were marked. Moreover, backend datasheets could be downloaded from the system for review (Figure 2E).

## 3 Results and Discussion

### 3.1 Proper Position for Wearing a Bluetooth Tag

**Figure 3:**
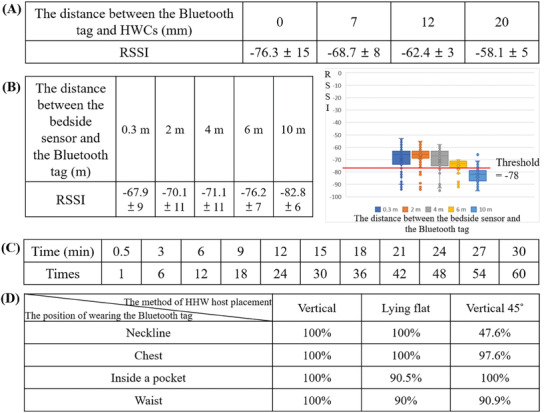
Results of the sensor evaluations. A) The RSSI strength when the Bluetooth tag is worn at different distances from the body of the HWC. B) The RSSI strengths at different distances between the bedside sensor and the Bluetooth tag. The signal values are also shown in box‐and‐whisker plots. C) The number of times that a Bluetooth tag will be scanned within a given time duration at the chosen scanning frequency of the bedside sensor. D) Accuracy rates for different methods of HHW host placement and different wearing positions of the Bluetooth tag.

The signal strengths of Bluetooth tags worn at different distances from the body were tested in this study. The RSSI value is only −76 ± 15 when the Bluetooth tag is worn directly against the body (i.e., at a distance of 0 mm), and the RSSI gradually increases to −68 ± 8, −62 ± 3, and −58 ± 5 when the distance is increased to 7, 12, and 20 mm, respectively (**Figure** **3**A). Thus, the results showed that packaging materials with a 20 mm distance were the best choice for use in subsequent research. A previous study^[^ ^35^ ^]^ reported that a Bluetooth signal is affected by the media it passes through, among which water, fat, and muscle, as components of the human body, have the most significant impact. These results are consistent with the present research. When the tag is worn at a distance of 20 mm from the body, less of the signal is absorbed, resulting in a signal intensity that is double its original value.

### 3.2 Bedside Sensor Signal Reception Test

In the developed system, a Bluetooth signal receiver in a ward is an IoT device that receives the Bluetooth tag signals worn by HCWs to confirm when HCWs are in the ward. Before beginning the clinical trials, a signal reception test of the bedside sensor was conducted in a 25 × 50 m^2^ room. Distances of 0.3, 2, 4, 6, and 10 m between the Bluetooth tag and the bedside sensor were tested, and the corresponding average and standard deviation of the RSSI values were found to be −67.9 ± 9, −70.1 ± 11, −71.1 ± 11, −76.2 ± 7, and −82.8 ± 6, respectively (Figure 3B). The farther the Bluetooth tag is from the bedside sensor, the lower the RSSI will be, providing the potential to estimate the distance between an HCW and the bedside sensor.

At the clinical site where this study was conducted, every ward is an open ward with a length of 380 cm. From the end of the ward, the movable workbench in front of the ward is placed at a distance of ≈440 to 470 cm, the mobile nursing cart is placed at ≈470–500 cm, and the nursing station is at ≈760 cm. Therefore, ideally, the RSSI should enable the distance from a Bluetooth tag to the bedside sensor to be distinguished to within 4 m. However, as seen from box‐and‐whisker plots of all the RSSI values, the RSSI cannot be used to distinguish between signals originating from closer or farther away than 4 m. Only an RSSI value less than −78 can be considered to indicate a distance greater than 6 m. Therefore, the bedside sensor can barely distinguish when an HCW is in the ward or working at a mobile nursing cart. Other studies have shown that using the RSSI values of Bluetooth signals to determine the indoor distance from a single Bluetooth signal receiver results in errors of 100–200 cm.^[^ ^36^, ^37^, ^38^ ^]^ Therefore, the Bluetooth signal receiver used as the bedside sensor in the developed system can barely identify the locations of HCWs and when they are in the corresponding ward.

Accordingly, to more reliably confirm when HCWs are present in the ward, the scanning frequency of the bedside sensor was increased to achieve greater accuracy. The frequency of scanning was changed from once every 3 min to once every 30 s. The results showed that the bedside sensor has high stability when scanning and receiving Bluetooth signals. Therefore, instances in which HCWs pass through a ward and enter the ward only briefly to check the patient's condition can be filtered out (Figure 3C).

### 3.3 Evaluation of the HHW Detection Device

The HHW detection device is the core device in the developed system. The results obtained with different wearing positions of the Bluetooth tag, with different orientations of the host, with different orders of ultrasonic sensors, and in testing under different noise interference conditions are presented in this section.

The optimal positioning of the ultrasonic sensor and the accuracy of the HHW host's Bluetooth receiver are related to the position where the HCW is standing while washing their hands. This is because ultrasonic sensors detect distance by sending and receiving ultrasonic echoes, and the distance between a Bluetooth signal emitter and the receiver can affect the corresponding signal strength. It is essential that the ultrasonic waves transmitted by the ultrasonic sensor can be smoothly reflected back to the receiver after coming in contact with the HCW. Therefore, the ultrasonic sensor is installed above the faucet. When an HCW approaches the sink to wash their hands, an ultrasonic sensor in this position can reasonably accurately receive the echoes reflected by the clothing on the chest and abdomen of the HCW. Previously, several optical distance sensors, including infrared and laser sensors, have been tested. However, several factors, such as the surface roughness and color of the target object, can affect the accuracy of such an optical device, making correct data challenging to obtain. The use of the RSSI value of the Bluetooth signal was also considered to determine the distance between the HCW and the sink. However, the results were not ideal because wireless signals are more susceptible to environmental interference and influence. Finally, it was concluded that mechanical measurements using ultrasonic waves offer greater reliability.

Nevertheless, the Bluetooth tag signal of an HCW who is washing their hands should also be detected by the Bluetooth receiver in the HHW host while the ultrasonic sensor is operating. Therefore, a cross‐experiment was performed to test the accuracy of signal reception depending on the placement of the HHW host and the wearing position of the Bluetooth tag. On the basis of the position of the antenna of the HHW host, three possible host placement methods exist: vertical, lying flat, and vertical at 45°. Considering the habits of HCWs, four wearing positions were considered for the Bluetooth tag: worn at the neckline, on the chest, inside a pocket, and at the waist. The results for signal detection accuracy show that when the host is placed vertically, the Bluetooth signal reception accuracy is 100% when the Bluetooth tag is worn on the chest or at the neckline, whereas the accuracy is lower when the host is placed vertically at 45° (Figure 3D). Therefore, the HHW host should be placed vertically, and the Bluetooth tag should be worn on the front of the chest.

**Figure 4:**
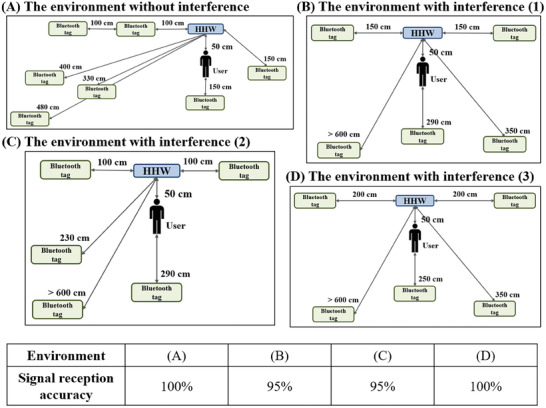
Signal reception accuracy in different environments. A) An environment without interference. B) An environment with interference of type (1). C) An environment with interference of type (2). D) An environment with interference of type (3).

At least eight HCWs wearing Bluetooth tags will typically be present simultaneously in the clinical environment. To ensure the proper functioning of the Bluetooth signal receiver in the HHW host, the accuracy in the presence of multiple Bluetooth tags was tested when a specific Bluetooth tag was 50 cm away from the HHW host and other tags were 100 to 600 cm farther from the HHW host. In this scenario, the Bluetooth tag that is 50 cm away represents a handwashing HCW. Thus, its MAC address should be recognized by the HHW Bluetooth receiver. The results show that in a simple signal environment without any other interference, the presence of other Bluetooth tags placed at least 100 cm away from the HHW host does not affect its accuracy, as indicated by the observed accuracy of 100%. However, in a complex signal environment with interference from other electrical devices, other Bluetooth tags should be at least 200 cm away from the HHW host to ensure 100% accuracy; when other Bluetooth tags are present at a distance of 150 or 100 cm, the accuracy can be reduced to 95% (**Figure** **4**).

**Figure 5:**
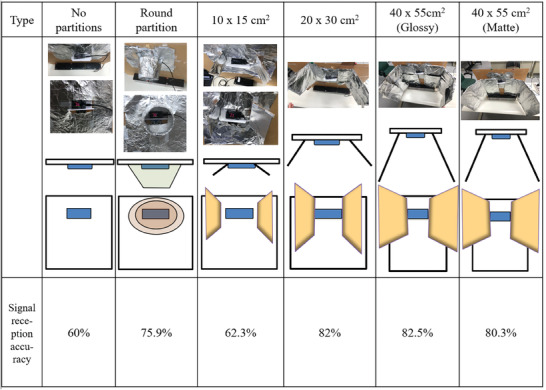
In a Wi‐Fi signal enhancement context, a study was conducted to compare the signal reception accuracy of different partition types. The control setup included a router with no partitions, resulting in a 60% signal reception accuracy. Other setups included a round partition, a 10 × 15 cm^2^ partition, a 20 × 30 cm^2^ partition, and two 40 × 55 cm^2^ partitions with different surface finishes, glossies and mattes. Each setup was accompanied by a corresponding image, a schematic representation, and a signal reception accuracy percentage. The highest signal accuracy achieved was with the glossy 40 × 55 cm^2^ partition at 82.5%, whereas the round partition also showed a significant increase to 75.9% compared with the control setup.

In the actual clinical environment, the mobile nursing cart and movable workbench at the ward's entrance are likely to be placed within 150 cm of the HHW host. There is some possibility that the HHW host will not receive the data correctly when an HCW is washing their hands while other Bluetooth tag carriers are working at the mobile work cart and movable workbench. Therefore, the adoption of metal partitions was considered to increase the accuracy of the HHW host receiver. For testing, partitions with different cross‐sectional shapes and sizes were installed, the correct Bluetooth tag was placed 40 cm away from the host, and potentially interfering Bluetooth tags were placed 50 cm away. The results show that the accuracy of the host in receiving the correct Bluetooth tag is somewhat increased with the installation of a partition, but the difference is insignificant (**Figure** **5**). Therefore, these partitions are not useful for improving signal accuracy in actual clinical practice.

**Figure 6:**
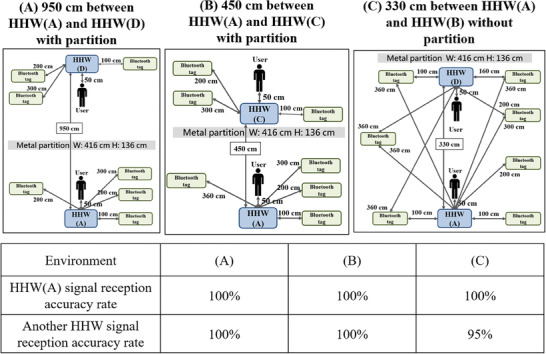
Interference between two HHW hosts. A) The distance between the two HHW hosts is 950 cm, and there is a partition between them. B) The distance between the two HHW hosts is 450 cm, and there is a partition between them. C) The distance between the two HHW hosts is 330 cm, and there is no partition between them.

Since the wards at the experimental site are composed only of simple wooden cubicles, two adjacent HHW hosts could interfere with each other. The presence of decorative material in wards does not attenuate Bluetooth signals. To test the accuracy of two nearby devices, two HHW detection devices were placed at distances of 950, 450, and 330 cm, and 9 Bluetooth tags were deployed: two tags representing the handwashing users to be detected and seven tags representing environmental noise. The results show that detection errors may occur when there are potentially interfering Bluetooth tags within 100 cm from an HHW host and when the distance between the two HHW hosts is 330 cm; during testing in this scenario, the accuracy of one HHW host decreases by 95%. For the other distance settings, the Bluetooth tag detection accuracy was 100% (**Figure** **6**). Notably, the distance between two sinks in adjacent wards in the actual clinical environment is only 350–420 cm, which implies that there is a finite probability that an HHW host will detect the MAC address of the wrong Bluetooth tag.

The stability of the system is an essential concern. Initially, a Raspberry Pi 4B combined with a 3.5‐inch RPi LCD was tested as the HHW host. The Raspberry Pi 4B single‐board microcomputer was responsible for processing the dynamic display on the LCD, Bluetooth tag sensing, and uploading data to the cloud database through Wi‐Fi. A simple RP2040‐Zero single‐chip microcontroller paired with an HC‐SR04 ultrasonic sensor was used to measure the distance. However, after a long period of continuous power‐on testing, the Raspberry Pi‐based HHW detection device could no longer detect the presence of Bluetooth tags. This problem can be solved only by manually restarting the device. Therefore, the HHW detection device was changed to the current version. In addition to the Watchdog timer, a remote power controller and rated control programs were added to restart the HHW device automatically. In the case of a severe problem, the remote power controller can also be used to reboot the device manually to enhance the system's stability.

### 3.4 Evaluation of the HHA Detection Device

The main detection unit of the HHA detection device is a pressure sensor. In the HHA detection device, no sensor is responsible for detecting a specific user. According to a survey of nurses' working habits and the established hand hygiene standards at the Taoyuan Hospital of the Ministry of Health and Welfare, at least 2 ml of alcohol‐based hand sanitizer is considered required to meet hygiene standards in HHA. Therefore, the mechanism design of the plate placed under the sanitizer spray bottle and the pressure applied by users were studied to facilitate the detection of the dispensing of sufficient sanitizer.

**Figure 7:**
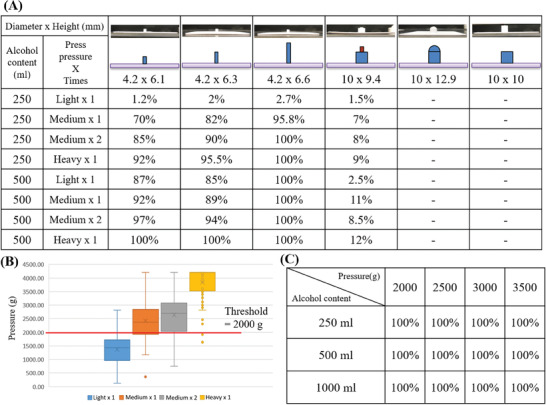
Cross‐testing was performed with different shapes of pressure plate contact mechanisms, different sanitizer volumes, and different pressure levels. A) Probabilities of induction with various forms of pressure plate contact mechanisms, different sanitizer volumes, and different pressure levels. B) Box‒and‐whisker plots of the quantitative pressing forces (in grams) measured under four different subjective pressure levels to determine the pressing force threshold. C) Rates of pressure plate activation under different pressing forces at different remaining sanitizer volumes, confirming that a pressing force threshold of 2000 g is appropriate.

The pressure testing element of the HHA detection device is a spring‐operated pressure plate installed below the sanitizer spray bottle. Therefore, the amount of remaining sanitizer in the bottle and the length and shape of the contact mechanism between the pressure plate and the pressure test element were the focus of testing. Cross‐testing was performed using different shapes of pressure plate contact mechanisms, different sanitizer volumes, and different levels of applied pressure. The results revealed a 100% induction probability when pressing a plate contact mechanism with a diameter of 4.2 mm and a height of 6.6 mm, excluding light pressing and one‐time medium pressing. However, when the contact mechanism was shortened, the pressure sensor could not be activated 100% of the time for signal transmission unless the user increased the pressure. When the diameter of the contact mechanism was increased to 10 mm, regardless of the contact shape of the front end, the pressure sensor lost its function (**Figure** **7**A).

After the contact mechanism of the plate was confirmed, eight non‐HCWs were asked to press the dispenser of the spray bottle mounted on the pressure sensor at four different pressure levels. A scale was used to record the pressure, and data were collected to construct box‐and‐whisker plots to determine the threshold of the necessary pressing force (Figure 7B). The results showed that the handwashing detection device could be activated 100% of the time when the pressing force was greater than 2000 g (Figure 7C). Thus, the threshold value for the HHA detection device was set to 2000 g, which is the general pressure applied under the subjective intentions of a user without extra attention given to squeezing out at least 2 ml of hand sanitizer.

### 3.5 Sandbox Text of the Handwashing Detection System

According to previous research,^[^ ^39^ ^]^ among hand hygiene experiments conducted in 801 clinical environments, only 72 of the selected cases represented adult ICU environments, such as the one that is the focus of this study. Owing to the heavy workload in adult ICU environments, a sandbox test should be conducted to ensure the usability of a system in such an environment before it is actually deployed to monitor hand hygiene behaviors in clinical practice.

**Figure 8:**
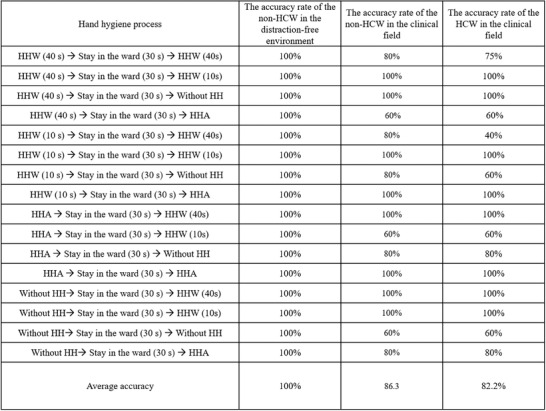
Accuracy rates for 16 possible basic hand hygiene behaviors in sandbox simulation tests conducted in different environments by non‐HCWs and HCWs. The processes involve various combinations of handwashing (HHW) for 10 or 40 s, hand antisepsis (HHA), and periods of staying in the ward for 30 s, with or without hand hygiene (HH) steps. The table records accuracy rates for these behaviors in a distraction‐free environment, a nonclinical field, and a clinical field for both non‐HCWs and HCWs. The average accuracy across all tested behaviors was 100% for non‐HCWs in a distraction‐free environment, 86.3% for non‐HCWs in the clinical field, and 82.2% for HCWs in the clinical field. This suggests that the complexity of the environment may affect adherence to hand hygiene processes, particularly for HCWs.

In this study, all 16 possible basic hand hygiene behaviors were identified, and sandbox simulation tests were conducted in different environments (laboratory and clinical) and with different types of users (non‐HCWs and HCWs). One handwashing sandbox test was performed in the laboratory with non‐HCWs and in an environment without interference, and the identification accuracy rate was 100%. When non‐HCWs were asked to perform the 16 handwashing behaviors in an ICU environment, the identification accuracy decreased slightly to an average rate of 86.3%. When HCWs were asked to perform the same test, the average identification accuracy rate was 82.2% (**Figure** **8**).

These results might be related to environmental issues and the behaviors of different users. In the laboratory environment, the computer was the only machine with potentially interfering effects. However, a large amount of medical equipment that can generate electric fields is present in the ICU. Furthermore, in the ward environment, Bluetooth signals may echo badly, and these effects may be difficult to alleviate.

In addition, particular handwashing behaviors of the user—for example, the standing location, the gestures of the hands, or the hand positions—could affect the detection results of the system. Accordingly, an analysis of the possible reasons for system errors shows that for more than 73.8% of HHW actions, the results cannot be continuously detected because the specified time or noise is detected from other Bluetooth tags. Because an ultrasonic sensor is used for detecting the handwashing time, the user's position can have a large effect. Changes in the user's position, the hands leaving the detection range for the application of soap, the material of the clothing, and other factors may cause the ultrasonic detector to be unable to sense the user. Thus, there are many reasons why HHW timing detection may be interrupted. Moreover, decorations in the ward can generate echoes of electromagnetic waves while only minimally blocking noise from other wards. The reason for the remaining 26.2% of the errors is speculated to be that the 4G signal from the telecommunication company is unstable, so data from the devices may not be uploaded to the cloud drive in real time.

### 3.6 Comparison of Actual Clinical Field Survey Data with Data from the Detection System

The HCWs who participated in the project included 15 nurses, specialist nurses, physical therapists, and occupational therapists from the adult ICU. The data collected by the developed system were compared with the manual audit data collected by hospital infection control nurses to determine the accuracy of the data collected when the system was used. The manual inspections were divided into three periods, two of which lasted from 9:30 to 10:30 during the morning and the other from 2:30 to 4:30 in the afternoon. The total duration was 4 h. The manual audit data were divided into three parts: the time of entering the ward, the time of leaving the ward, and the handwashing time (HHW or HHA). After this division of events, the number of data points increases from 51 to 112. The number of data points arriving inside/leaving wards is 52, the HHW is 36, and the HHA is 29. Whereas the number of data points detected by the system after noise was filtered out was 156, the number of data points that matched the actual audit events was 81, and the data points arriving inside/leaving the wards, HHW, and HHA were 28, 24 and 29, respectively. The number of data points that were not correctly mapped was 77.

According to formula (1), the accuracy of the whole system in this study was 72% (81/112 = 0.723). To further analyze which devices were the causes of the detection errors, the accuracies of the individual detection devices were calculated. The accuracy of the bedside sensor, which was calculated via formula (2), was 53.8% (28/52 = 0.538). Formulas (3) and (4) were used to calculate the accuracies of the HHA and HHW detection devices, which were 100% (29/29 = 1) and 67% (24/36 = 0.667), respectively. Finally, the noise rate of the developed system, which was calculated via formula (5), was found to be 49% (77/156 = 0.493).

After further discussion of the reasons why the system could not fully adapt to the actual situation, it was found that the system performance was affected not only by the handwashing habits of users but also by the decorations and other mechanical noise in the clinical environment. It was discovered that decorations in the clinical area amplified the Bluetooth signal so that the bedside sensor could receive the signal from a Bluetooth tag within 800 cm, and the signal strength could not be used for filtering. The distance to the workbench in front of the ward is ≈440 to 470 cm, the distance to the mobile nursing cart is ≈470–500 cm, and the distance to the nursing station is ≈760 cm from the bedside sensor. When HCWs are working in the above locations, the bedside sensor will also detect these nurses as being in the ward. This severely affects the accuracy of bedside sensors, increasing noise due to erroneous detection. In addition, the partitions between two adjacent wards are not sufficient to block Bluetooth signal transmission. Therefore, during actual operations, when HCWs are working in the ward next door, at the workbench in front of the ward, at the mobile nursing cart, or sometimes even at the nursing station, the bedside sensor can detect those HCWs and consider them to be in the ward. It cannot fully determine whether HCWs are performing nursing work in the ward or performing paperwork outside the ward, which is consistent with the test results of Experiment 3.2.

The decoration issues and the noise from medical machines in the clinical environment also affect the HHW host, which also receives Bluetooth signals. Bluetooth signals from other wards or locations, such as the workbench, the mobile nursing cart, or the nursing station, can also sometimes be received by the HHW host. This interference among Bluetooth tags prevents the HHW detector from detecting the correct Bluetooth tag signal. However, when data with no corresponding detection of the Bluetooth tag from the HHW detector are rejected, the accuracy of the HHW detection device is 100%. This indicates that the HHW timing detection of the HHW device is correct. The data from Experiment 3.3 also confirm these test results.

Previous research^[^ ^36^, ^37^, ^38^, ^40^ ^]^ has shown that when a handwashing detection system does not use close‐range RFID technology (which requires swiping ID cards or closely approaching the RFID reader), cameras, or other devices, the need to pinpoint whether an HCW is present in the ward is one of the practical limitations of such a system. Those studies also reported that when a single RFID detection device is used, the performance in a simulated environment is excellent. However, the performance of such systems is still poor in actual applications. The accuracy of detecting whether an HCW enters or exits a ward is only 54.3%.^[^ ^41^ ^]^ This accuracy is only slightly higher than that achieved in the present study via BLE technology. This accuracy is only slightly higher than that achieved in the present study using BLE technology, and the RFID acceptors need to be installed above the door; when the environment is an open ward, RFID could be a research limitation.

### 3.7 Limitations of Handwashing Detection System Devices in Clinical Application

The system developed in this research adopts IoT technologies to achieve active handwashing detection. The main purpose of this study is to overcome the limitations of traditional direct observation methods. However, the system faces several constraints in clinical applications.

First, the hardware limitations are explained. The ultrasonic module in HHW may be interrupted by clothing materials or standing angles. The Bluetooth signal received by bedside sensors is affected by signal reflection, noise, and interference, reducing accuracy in detecting ward entry and exit. Moreover, various limiting factors, such as funding, the implementation environment, the workforce, and time costs, may affect the scope of device application at medical sites, in addition to considerations such as whether the adopted devices affect the work of onsite HCWs. In terms of technical aspects, current technological developments in related fields are limited, such as the positioning accuracy of Bluetooth technology, the size and weight of devices, the battery capacity and weight, the time required for charging, and the time for which a device can be used. There is also mutual influence among the abovementioned limiting factors. For example, funding affects the price and quantity of devices that can be selected, the size and weight of a device affect the user's willingness to wear it, and the implementation environment affects the size of the devices that can be used.

Second, there is another challenge in terms of system stability. This study revealed that if an IoT device is turned on for a long time, it will have a chance of crashing and being unable to be restarted remotely by software.^[^ ^42^ ^]^ Therefore, each IoT device should be equipped with a remote power controller, and the software should be regularly relaunched to address related problems. In addition, the use of data from multiple types of sensors poses considerable challenges for data integration. Data received from sensors in different formats also produce noise and ambiguity, leading to competitive and conflicting errors. The research in^[^ ^43^ ^]^ suggested that increasing the amount of data is one possible solution for system reliability.

In addition to the limitations of the system, other factors affect the present research, such as a small sample size, the use of an ICU ward only, short‐term research, and environmental factors. The generalizability of the results may not be clear. The psychological state of HCWs is another main factor affecting the present research. The time required to receive a case and the context of data collection both affect whether an HCW cooperates with electronic devices for handwashing detection. Many studies have shown that the longer HCWs are asked to wear electronic tags for handwashing detection, the worse their effectiveness is over time.^[^ ^44^, ^45^ ^]^ The present project also has similar problems. Within approximately half a year after the HCWs at Taoyuan Hospital were asked to wear Bluetooth tags for this study, HCWs started to fail to wear Bluetooth tags correctly, forget to wear Bluetooth tags at all, forget to charge their Bluetooth tags, or damage their Bluetooth tags and consequently stop being monitored, increasing the difference between the number of cases collected during manual inspections and by the electronic monitoring equipment.

However, this project contributes to both hand washing and hand hygiene detection. It involves technological innovation, which combines multiple technologies, such as Bluetooth positioning, ultrasonic sensing, and pressure sensing, to provide a comprehensive handwashing monitoring solution. Real‐world application testing has also been adopted and provides detailed accuracy data, which is valuable for understanding how the system will perform in a real‐world setting. Moreover, the automation and long‐term monitoring of hand hygiene compliance and accuracy have the potential for long‐term monitoring, which could overcome some of the limitations of traditional direct observation methods.

## 4 Conclusion

Hand hygiene is vital for preventing HAI, but traditional direct observation methods have limitations. To overcome these limitations, a handwashing detection system was developed using BLE and wireless technology. It combines IoT technology to determine the locations of HCWs with an active monitoring system to detect their hand hygiene status. The accuracy of this project was evaluated before its actual application in the adult ICU, and the accuracy was 100% when it was used in an environment without interference. In a clinical environment, the accuracy was 82.2%, and in actual usage in the adult ICU, it was 72% due to many sources of interference, user behaviors, and environmental limitations. The main influencing factor was environmental limitations, and the accuracy of HCWs was only 53.8%. Improving the positioning technology for HCWs will improve the accuracy of the whole system. The system can automatically calculate hand hygiene compliance and accuracy rates through active monitoring without consuming considerable human resources. This method can potentially reveal the hand hygiene performance of HCWs through long‐term detection and overcome the disadvantage of direct observation of hand washing behavior. To our knowledge, this is the first IoT system that simultaneously monitors both HHW and HHA practices while providing real‐time data visualization and analysis, representing a significant advancement in hand hygiene monitoring technology. However, further research is needed to evaluate the impact of the system on improving medical care and reducing HAI incidence. Additionally, comparative studies are needed to determine its effectiveness relative to other hand hygiene monitoring methods. In summary, the developed handwashing detection system has the potential for detecting hand hygiene behavior and can automatically calculate hand hygiene compliance and accuracy rates. As positioning technology for HCWs is improved, the system's accuracy could also be enhanced, which may help address some issues associated with direct observation of handwashing behavior. However, more research is needed to determine the system's effectiveness in HAI prevention.

## Conflict of Interest

The authors declare no conflict of interest.

## Supporting information



Supporting Information

## Data Availability

The data that support the findings of this study are available from the corresponding author upon reasonable request.
