# Supplementary material for: IoT‐Based Hand Hygiene Compliance Monitoring System and Validation of Its Effectiveness in Hospital Environments
Source: Glob Chall. 2024 Nov 12;8(12):2400124. doi: 10.1002/gch2.202400124 (PMC11637777; doi:10.1002/gch2.202400124)
Supplement: Supplementary file 1 — Supporting Information [file GCH2-8-2400124-s001.docx]

# Supplementary Methods

**IoT-Based Hand Hygiene Compliance Monitoring System and Validation of Its Effectiveness in Hospital Environments**

## **Handwashing timing and steps**

The Five Moments for Hand Hygiene recommended by the World Health Organization (WHO) are shown in **Supplementary Figure 1A**. Those five moments are before contact with patients, before any operations that require cleaning or sterility, after contact with patients' body fluids, after contact with patients, and after contact with the surrounding environment of patients. The main reasons are to prevent healthcare-associated infection (HAI) by preventing pathogens present on healthcare workers (HCWs) from entering the bodies of patients, being transmitted to other places, potentially spreading to other patients or the environment, or being carried to other sites [1].

***1.1 Protocols for performing hand hygiene with soap and water (HHW)***

The protocols used for hand hygiene with soap and water (HHW) used in this study were taken from the WHO Guidelines on Hand Hygiene in Health Care. There are 11 steps in the HHW procedure, as shown in **Supplementary Figure 1(B)**. The whole process took 40 to 60 seconds. First, wet your hands with water, use enough soap to cover all hand surfaces, and then start washing your hands by rubbing your palms against each other. The right palm is placed on the back of the left hand with the fingers interlocked and rub, and vice versa; then, the hand is rubbed together with the palm with intertwined fingers. The backs of the fingers are subsequently rubbed against the opposite palm with the fingers interlocked. Next, hold the left thumb on the right palm and rotate and rub it, and vice versa. Then, press the five fingers of your right hand tightly against the palm of your left hand and turn your hands back and forth, and vice versa. Finally, after completing the handwashing action, the hand should be rinsed with water, the hand should be dried with a disposable towel, and then a towel should be used to turn off the faucet [1].

The main products used for HHW are soaps; liquid, bar, and powder soaps are all acceptable. The typical soap in the medical field is liquid soap [1]. In Taiwan, hand hygiene products used in HHW must comply with relevant laws and regulations. For example, antiseptic hand hygiene products must comply with the Pharmaceutical Affairs Act. Medical soaps are commonly used for specific medical needs, and their ingredients include antibacterials, antifungals, antivirals, and moisturizers [2]. Antibacterial products must abide by the Cosmetics Hygiene and Safety Management Act. The "Table of Ingredients Usage and Limits of Antibacterial Agents in Cosmetics," published by the Ministry of Health and Welfare, includes 18 antibacterial agents. The antibacterial agents generally used in soaps are benzalkonium chloride, chlorhexidine gluconate, chloroxylenol, and 4-isopropyl-m-cresol [2, 3].

There are several considerations related to the soap chosen and the usage of the soap. It is desirable to select soaps with less irritating medical ingredients while abiding by relevant regulations and considering the interactions among soap, skin care products, and glove types. Moreover, soap should not be added directly to used containers. If a container needs to be reused, the container needs to be emptied, and then the interior and exterior surfaces of the container need to be washed with warm water, detergent, and an appropriate disinfectant. The container must subsequently be thoroughly dried, after which liquid soap can be added [1].

***1.2 Protocols for performing hand hygiene with an alcohol-based formulation (HHA)***

The protocols for hand hygiene with an alcohol-based formulation used in this research were also taken from the WHO Guidelines on Hand Hygiene in Health Care. There are eight steps in the HHA procedure, which take 20 to 30 s, as shown in **Supplementary Figure 1(C)**. First, approximately 2~5 ml of the alcohol-based formulation was used to fully cover all the palm surfaces, and the palms were rubbed against each other. The right palm was placed on the back of the left hand with the fingers interlocked and rubbing, and vice versa. Next, rub your hands together palm to palm with the fingers intertwined and then with the backs of the fingers against the opposite palm with the fingers interlocked. The left thumb on the right palm is subsequently held and rotated and rubbed, and vice versa. Finally, press the five fingers of your right hand tightly against the palm of your left hand and turn your hands back and forth, and vice versa. After completing the handwashing action, you wait for your hands to dry [1].

The standard products used for HHA in medical institutions include alcohol-based hand sanitizers with a total alcohol content of at least 70% v/v of ethanol, propanol, or isopropyl alcohol. Generally, alcohol-based hand sanitizers are available in liquids and gels. Hand sanitizers in gel form consist of not only alcohol but also thickeners to increase the viscosity of the product and emulsifiers to stabilize the gel. Regardless, alcohol-based liquids or gels need to be dispensed with a pump or sprayer [4]. In addition, when sanitizer needs to be added to a used container, the process is similar to that for adding soap to a used container; both disinfection and the use of a fully dry bottle are essential [1].

## **Sensors in the hand hygiene management system**

There are four types of detection devices in the system: bedside sensors, HHW detection devices, HHA detection devices, and Bluetooth tags. In the following paragraphs, the system components (**Supplementary Figure 2**), the characteristics of the devices, and the process of research and development are described.

The ESP32 DevKit, produced by Espressif Systems, is the core component of the bedside sensor. The ESP32 DevKit is a highly integrated single-chip microcontroller with Wi-Fi and Bluetooth capabilities, providing flexible and diverse communication options for IoT-related applications. The size of the bedside sensor is 25 × 78 mm^2^. It uses an ESP32-Wroom-32E as the processing unit, Bluetooth and Wi-Fi antennas as the communication units, and a USB interface as the power unit (**Supplementary Figure 3(A)**).

Two different hardware designs were considered for the HHW detection devices, which can accordingly be divided into first-generation devices and second-generation devices. The main difference lies in the choice of host. For both first-generation and second-generation equipment, an HC-SR04 ultrasonic sensor is used to calculate the distance. Second-generation HHW detection equipment was adopted in this research.

The first generation of HHW detection devices used the Raspberry Pi single-board microcomputer, which is popular in scientific education. The Raspberry Pi 4B was combined with a 3.5-inch RPi LCD, which meets the requirements of multitasking capabilities, low cost, small size, and easy installation and maintenance. The Raspberry Pi was responsible for processing the dynamic display on the LCD, Bluetooth tag sensing, and uploading data to the cloud server via Wi-Fi. A simple RP2040-Zero single-chip microcontroller was coupled with the HC-SR04 ultrasonic sensor to measure the distance of an object. The HC-SR04 sensor is widely used for noncontact distance measurements via ultrasonic waves. It calculates the distance to an object by emitting ultrasonic pulses and monitoring their reflection time. The function of the RP2040-Zero was to control the operation of the HC-SR04. The RP2040-Zero was responsible for converting electrical signals into actual distance values and transmitting those values to the Raspberry Pi board for interpretation through the USB interface.

Moreover, the RP2040-Zero could be skipped in the system, and the HC-SR04 could be directly connected to the GPIO of the Raspberry Pi, through which the sensor could be controlled and its data processed by the main program on the Raspberry Pi. However, a ready-made HC-SR04 program library is available on Arduino. Thus, ready-made resources were used in the first generation of HHW detection devices.

The first generation of HHW detection devices has many advantages, as mentioned above. However, there was one disadvantage that could not be overcome. After a long period of continuous power-on testing, the first-generation HHW monitoring system suffered unexpected errors in that it could not detect the presence of Bluetooth tags, which made the system unable to identify a specific HCW performing HHW. The Bluetooth driver of the Linux system on the Raspberry Pi, especially the BLE-related part, lost its function for unknown reasons after running continuously for some time. Currently, no corrective solutions, such as driver patching, are available to solve this problem. The only way to recover the system is to restart the device manually. This approach is not feasible; thus, other stable alternatives need to be found. Therefore, a second-generation HHW monitoring system was developed.

The second-generation HHW detection system consists of an embedded microprocessor system similar to that of the bedside sensing device. The LILYGO T-PicoC3 is used as the main module of the system and interfaces with the HC-SR04 ultrasonic sensor peripheral module, which is a multifunctional module that integrates RP2040 and ESP32-C3 chips and is equipped with a full-color LCD. The RP2040 provides high-efficiency processing capabilities with low power consumption and can handle the display of real-time information on the LCD screen. ESP32-C3 provides Wi-Fi and Bluetooth connections and is responsible for scanning and identifying Bluetooth tags and uploading data to the cloud through Wi-Fi. The LILYGO T-PicoC3 also has a watchdog timer. When the system stops for unpredictable reasons, Watchdog quickly and automatically resets the system; therefore, manual intervention is not necessary when the system fails. The size of the host is 28 × 52 mm^2^, and the size of the distance sensor is 20 × 80 mm^2^. The host uses an RP2040 ARM Cortex-M0+ as the processing unit, communication unit, and power unit and uses the same technology as the bedside sensor. The distance sensor used an HC-SR04 ultrasonic distance sensor as the sensing unit (**Supplementary Figure 3(B)**). Overall, the second-generation HHW detection system eliminates the problems of the first generation and simplifies the architecture of the entire system. The cost, volume, size, weight, and power consumption of the components are reduced, and the efficiency of the system is improved. Thus, the stability and reliability of HHW detection devices have been greatly improved.

The actions of users when pressing spray bottles are detected by an appropriate pressure-sensing component to monitor HHA. An ESP32 DevKit, which is the same microcontroller used in the bedside sensor, and an FSR 402 resistive film pressure sensor are used in the HHA detection device. The device additionally includes 3D-printed mechanical components that are designed to serve the required functions. The size of the host is 25 × 78 mm^2^, and the size of the sanitizer bottle under which the pressure sensor is placed is 85 × 95 mm^2^. FSR 402 serves as the sensing unit. The host uses an ESP32-Wroom-32E as the processing unit, and the communication unit and power unit also use the same technology as the bedside sensor (**Supplementary Figure 3(C)**).

Bluetooth tags based on BLE technology are used for personnel identification in this system. A DX-BT24A BLE module is embedded to serve as the BLE signal transmitter, along with a 200 mAh Li-Po rechargeable battery and a TP4056 Li-Po battery charger, and a heat-shrinkable tube is used as a basic package. The Bluetooth tag can be hung on a cable or ring from a clip on the clothes of HCWs. The size of the Bluetooth tag is 28 × 13 mm^2^. The DX-BT24A is used as the processing unit, the Bluetooth antenna is used as the communication unit, and the TP4056 charging module is used as the power unit (**Supplementary Figure 3D**).

## **The experimental environments in this study**

Two types of experimental environments were used for testing in this study: environments without electromagnetic wave interference and environments with other machines and other sources of interference. **Supplementary Figures 4(A**), 4(B), and 4(C) depict the experimental environments used to evaluate the HHW detection device to ensure that the Bluetooth receiver functions well. **Supplementary Figure 4(A)** shows a laboratory environment without interference, with only one HHW host and seven other Bluetooth tags at distances ranging from 100 cm to 490 cm. **Supplementary Figure 4(B)** shows an environment with interference, in which 5 other Bluetooth tags were used at distances ranging from 150 cm to more than 600 cm. However, the electromagnetic waves from the other computers cannot affect the Bluetooth receiver. **Supplementary Figure 4(C)** shows the scenarios used to test whether two HHW hosts in the environment with interference could influence each other when separated by distances of 330 cm, 450 cm, and 950 cm. **Supplementary Figures 4(D)** and 4€ show the sandbox test environments. **Supplementary Figure 4(D)** shows the clean environment in which non-HCWs were asked to perform handwashing behaviors to test the detection capabilities of the whole sensor system. Afterward test, the programming of the sensors was adjusted and fine-tuned. The clinical sandbox tests were conducted in the environment shown in **Supplementary Figure 4(E)**. In this environment, both HCWs and non-HCWs were asked to test the functioning of the system. After final tuning, the system could be used in the actual clinical environment, and the accuracy of the data from the sensors was evaluated through comparison with the data from manual inspection.

## **Architecture of the IoT software and data flow of the cloud server**

The network architecture of the handwashing detection system is shown in **Supplementary Figure 5**. The clinical environment is connected to the cloud through a 4G network. The collected IoT information is stored in a Raspberry Pi single-board microcomputer, and a virtual private network (VPN) is set up on the physical network. A backend program is used to load and analyze the data from the sensors and display the results on the frontend web page. Administrators can check the webpage of handwashing detection devices through the VPN to monitor the handwashing behaviors of HCWs.

The clinical data flow can be separated into six steps, as shown in **Supplementary Figure 6**. Each handwashing detection sensor among the IoT devices contains three different functional units: a sensing unit, a processing unit, and a communication unit. The first step of the data flow corresponds to the sensing unit of each device, which detects signals from different events and then transmits the raw data to the processing unit. After the data are processed, a signal is broadcast to the communication unit. The three types of IoT devices in the developed system are bedside sensors, HHW detection devices, and HHA detection devices. When the pressure sensor of the HHA detection device is activated, the ultrasonic sensor of the HHW detection device detects an object within 10 cm, or the bedside sensor receives a Bluetooth signal from a Bluetooth tag worn by an HCW. An HTTP connection is established between the IoT device and the Raspberry Pi 4B through a 4G signal. Then, the data can be uploaded to the cloud database via the class C private network domain through the 4G Wi-Fi router. A REST architecture and HTTP are used in this research. The raw data from the IoT devices are transmitted in accordance with the OpenAPI specification (OAS). After being received through an HTTP API written via the FastAPI web framework, the data can be stored in the cloud database. The next step is to transmit the clinical data through the VPN for storage in the database via the 6598 IANA-Reserved IPv4 Prefix for the shared address space established by the internet Engineering Task Force (IETF). The data can then be loaded and analyzed by the cloud server on the National Health Research Institutes (NHRI) intranet. The data after analysis can be displayed on the website. Finally, administrators can check the results on computers, mobile phones, or other display devices.

## **Information security in the handwashing detection system**

When transmitting data over the internet, various security risks should be considered, including data leakage, data tampering, identity theft, and malware. To ensure the security of the data transmission between the cloud server and the Raspberry Pi, a VPN is used in the developed system. Compared with the RFC6598 specification, the network address is in the IPv4 range reserved by the internet-Assigned Numbers Authority for shared address space in accordance with next-generation VPN implementation technology. Since 2020, the Linux kernel (version 5.6 and above) has included WireGuard as a built-in VPN. WireGuard uses powerful encryption technologies, including noise, Curve25519, ChaCha20, Poly1305, BLAKE2, SipHash24, and HKDF. Therefore, the data transmission process used in this study meets the information security level requirements specified by relevant laws and regulations.

## **Detailed process of clinical data compilation**

The data compilation process is divided into five steps: data preprocessing, handwashing period estimation, handwashing data recording, data analysis, and web page deployment (**Supplementary Figure 7**). After the raw data that need to be sorted are downloaded, data preprocessing is performed, data for which the period is too short or the Bluetooth MAC address is unknown are deleted, and the remaining MAC addresses are converted into Bluetooth tags for HCWs, HHW detection devices, HHA detection devices, and bedside sensors in the wards. The next step was to estimate the duration for which each HCW was in each ward from the available data. Since the bedside sensor transmits data every 30 seconds, the time errors for HCWs entering and exiting wards will be within 30 seconds. In accordance with the hand hygiene regulations formulated by the hospital, medical staff need to wash their hands within 3 minutes before coming in contact with patients or the environment. Therefore, after bedside sensor data are used to determine when an HCW has entered and exited a ward, a corresponding handwashing record for HHW can be stored if such a record is present, including the Bluetooth tag of the HCW, the room number of bedside sensors, and the duration of HHW. The data can be stored only when all three types of sensing data are available. After the number of handwashing events and the timestamps, Bluetooth tag signals, and other relevant information are recorded, the hand hygiene compliance and accuracy rates can be calculated, and the relevant information can be displayed on the web page.

# Supplementary Figures


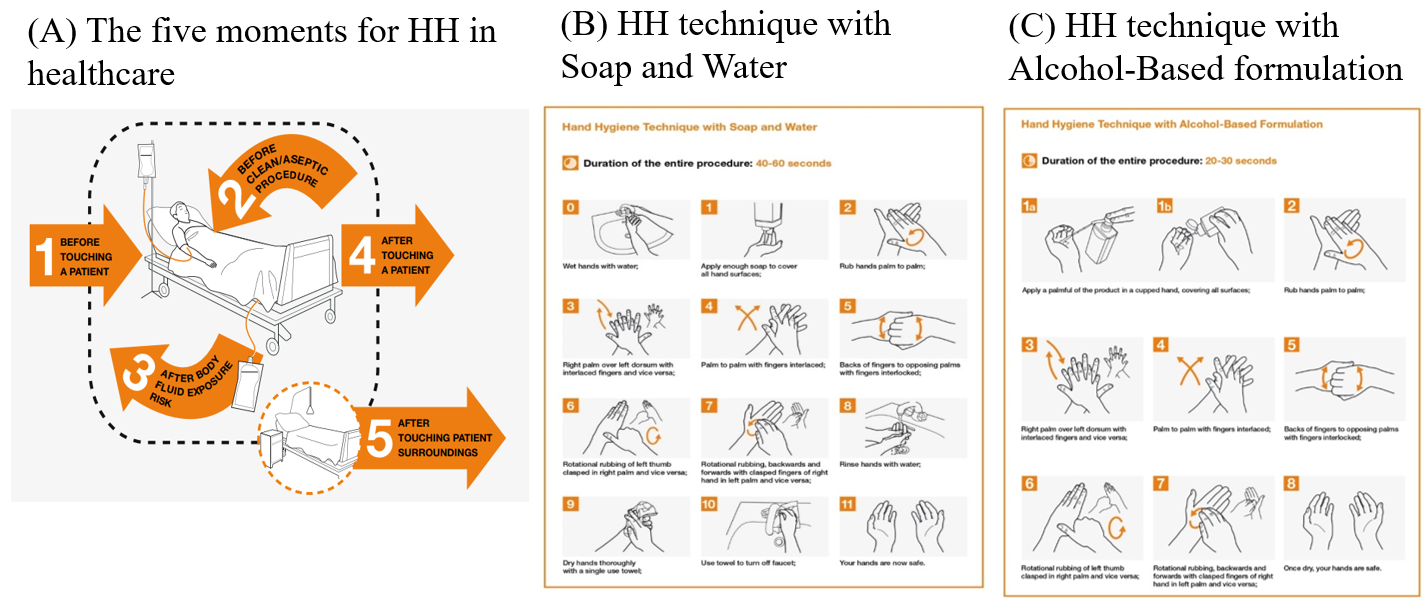


**Supplementary Figure 1.** Handwashing timing and steps (A) The Five Moments for Hand Hygiene recommended by the WHO. (B) WHO's guidelines for the HHW action. (C) The WHO's guidelines for HHA action.


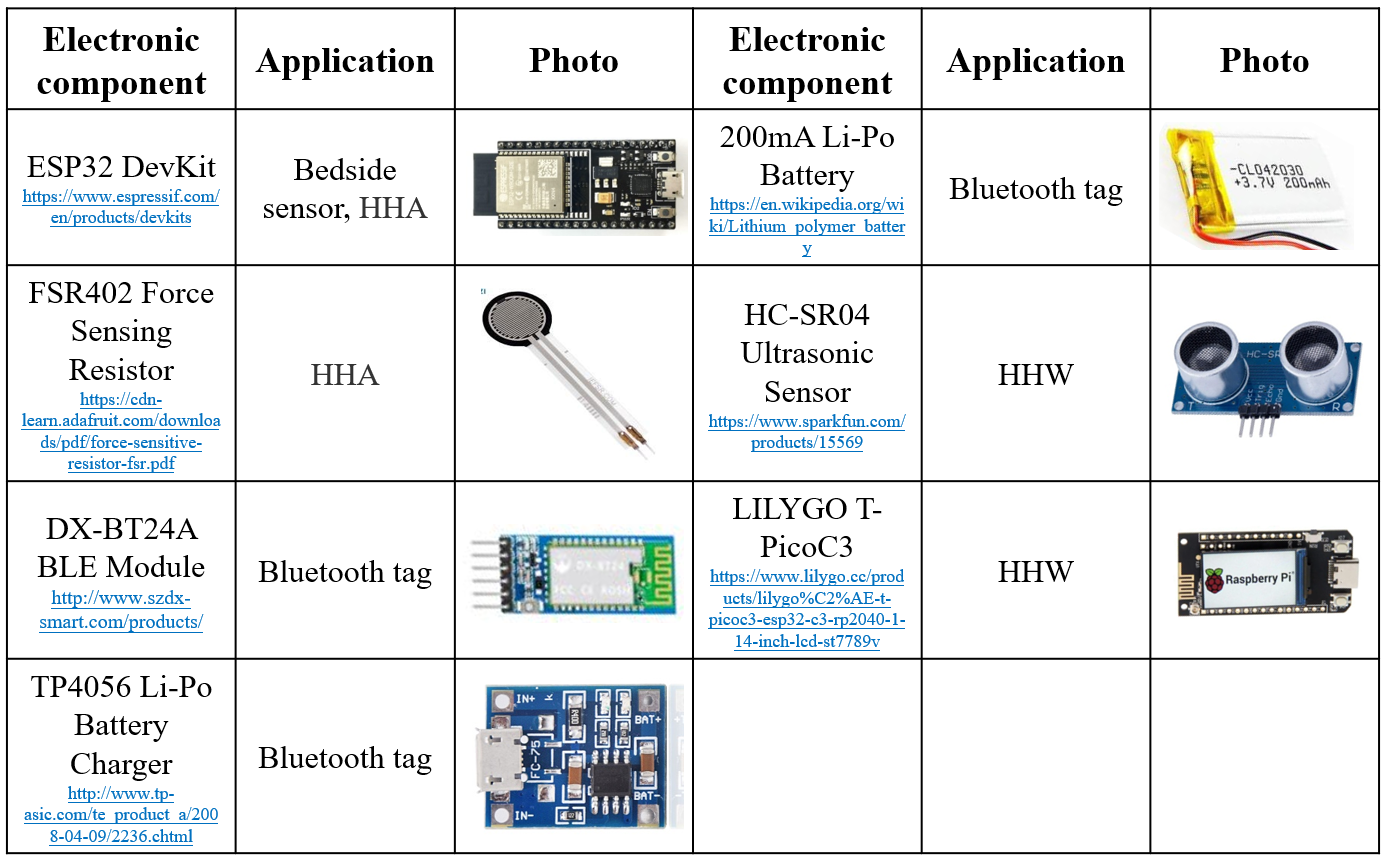


**Supplementary Figure 2.** The components used in the handwashing detection system.


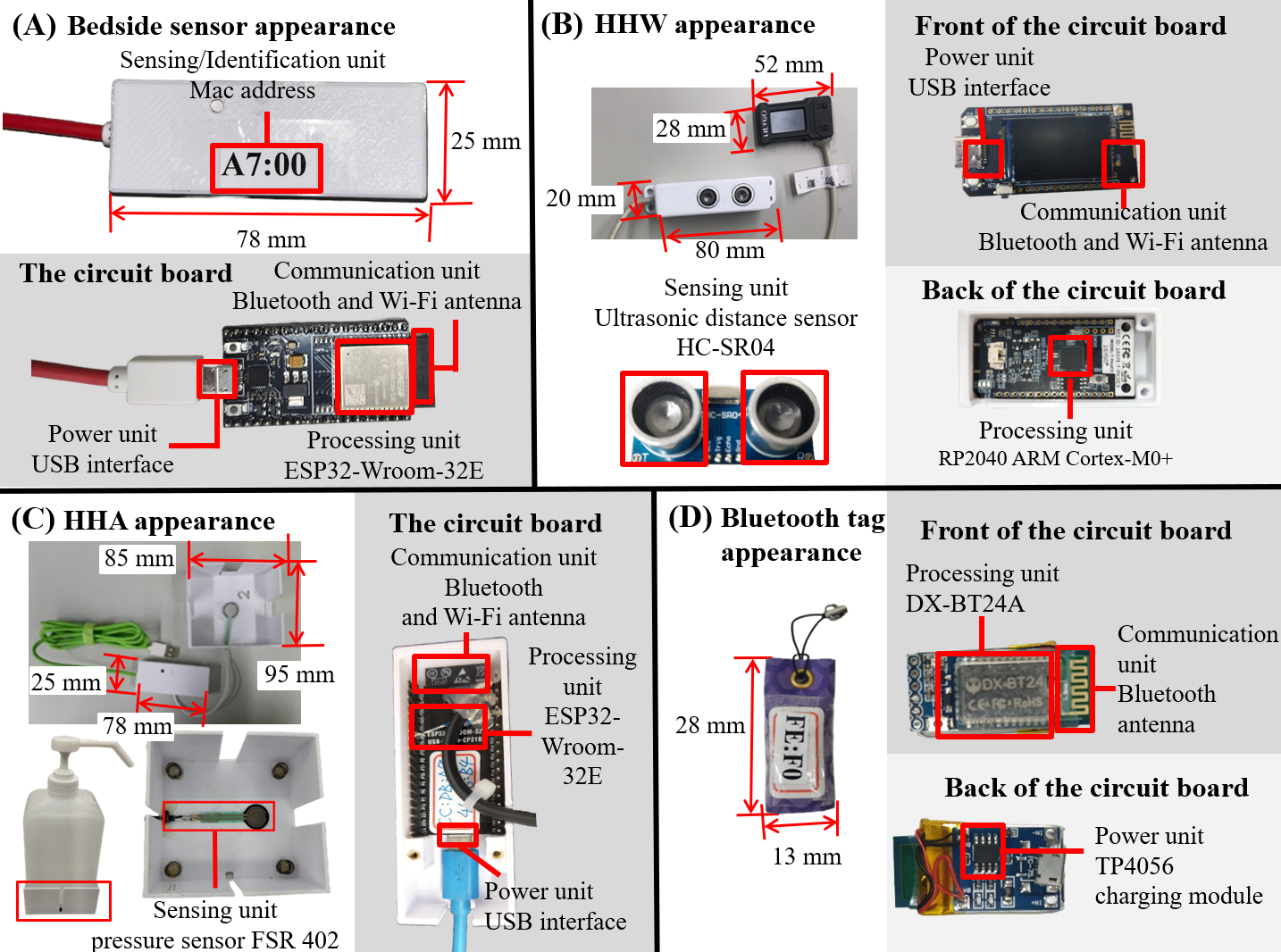


**Supplementary Figure 3.** Sensor sizes and components used in the handwashing detection system. (A) The components of the bedside sensor. (B) The components of the HHW detection device. (C) The components of the HHA detection device. (D) The components of the Bluetooth tag.


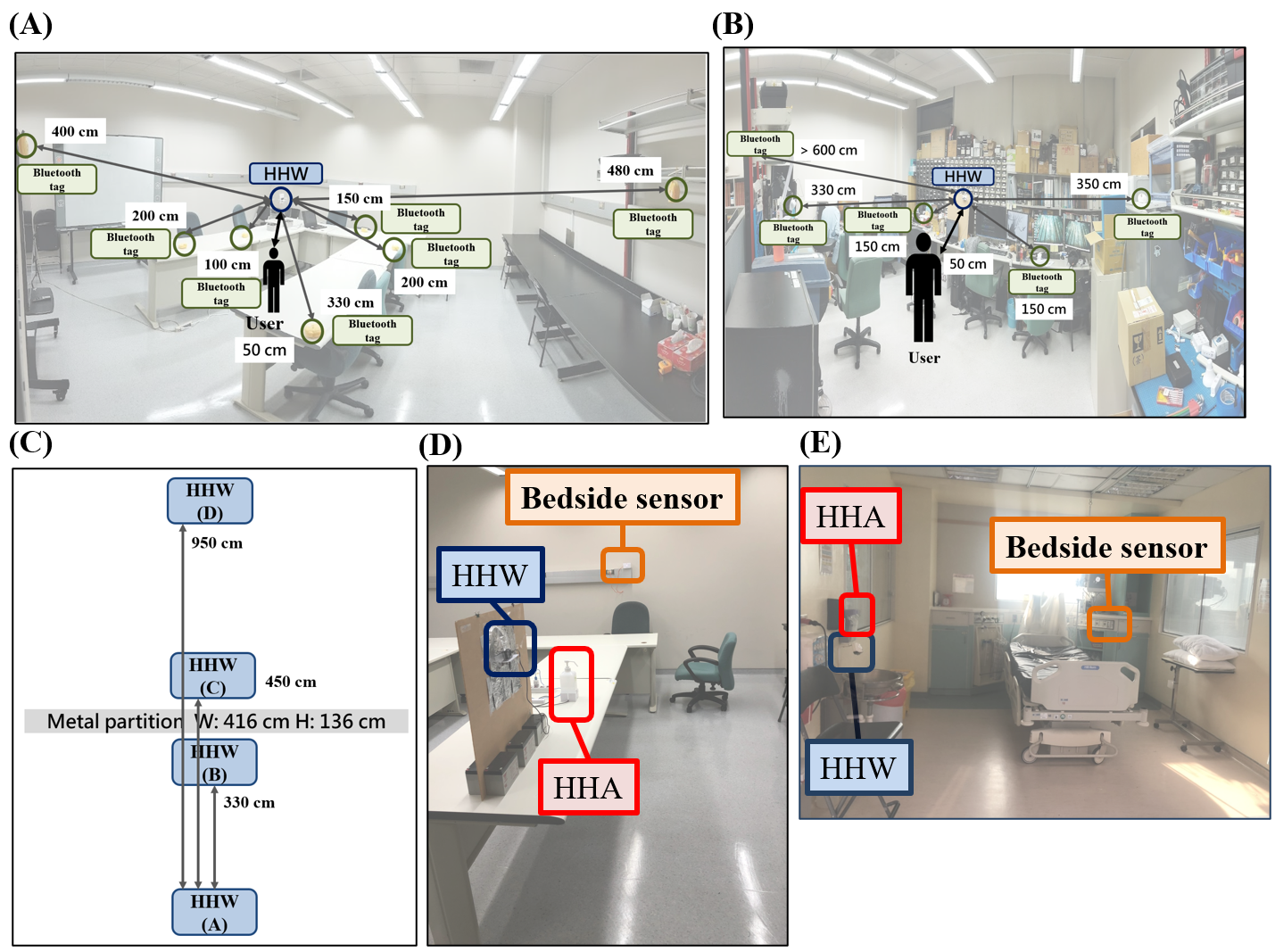


**Supplementary Figure 4.** Sensor test environments. (A) One HHW host was tested by non-HCWs in an environment without interference. (B) One HHW host was tested by non-HCWs in an environment with interference. (C) Two HHW hosts were tested by non-HCWs in an environment with interference. (D) Sandbox tests were performed by non-HCWs in an environment without interference. (E) Sandbox tests were performed by both non-HCWs and HCWs in a clinical environment.


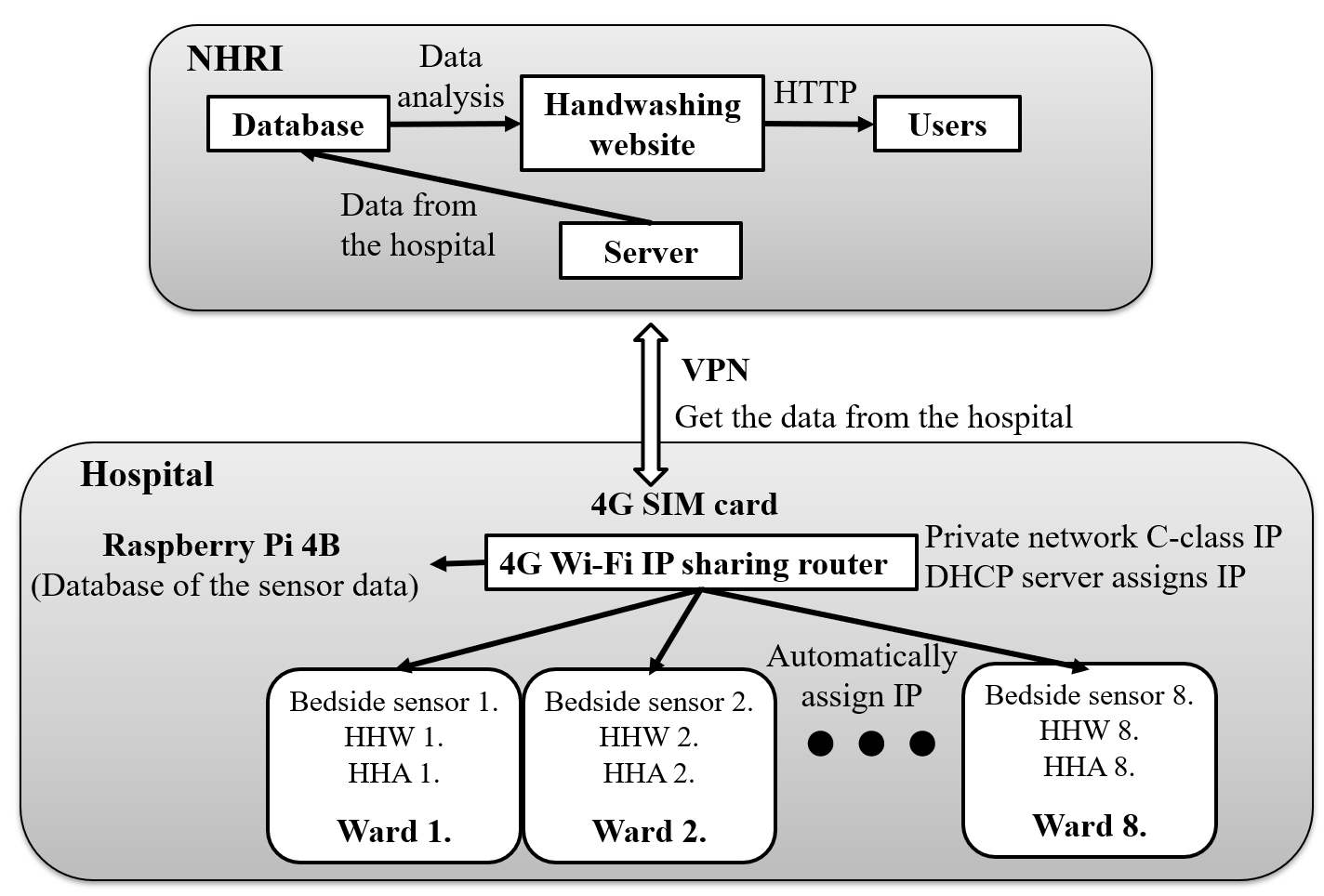


**Supplementary Figure 5.** Architecture of the IoT software. Every sensor in the handwashing detection system in the wards has a Wi-Fi communication unit, and a 4G SIM card is used to collect information for storage in a Raspberry Pi 4B. A virtual private network (VPN) is used to send the data to the cloud server and the database via the intranet of the National Health Research Institutions (NHRI), and the data can be analyzed and displayed to users on a website through HTTP.


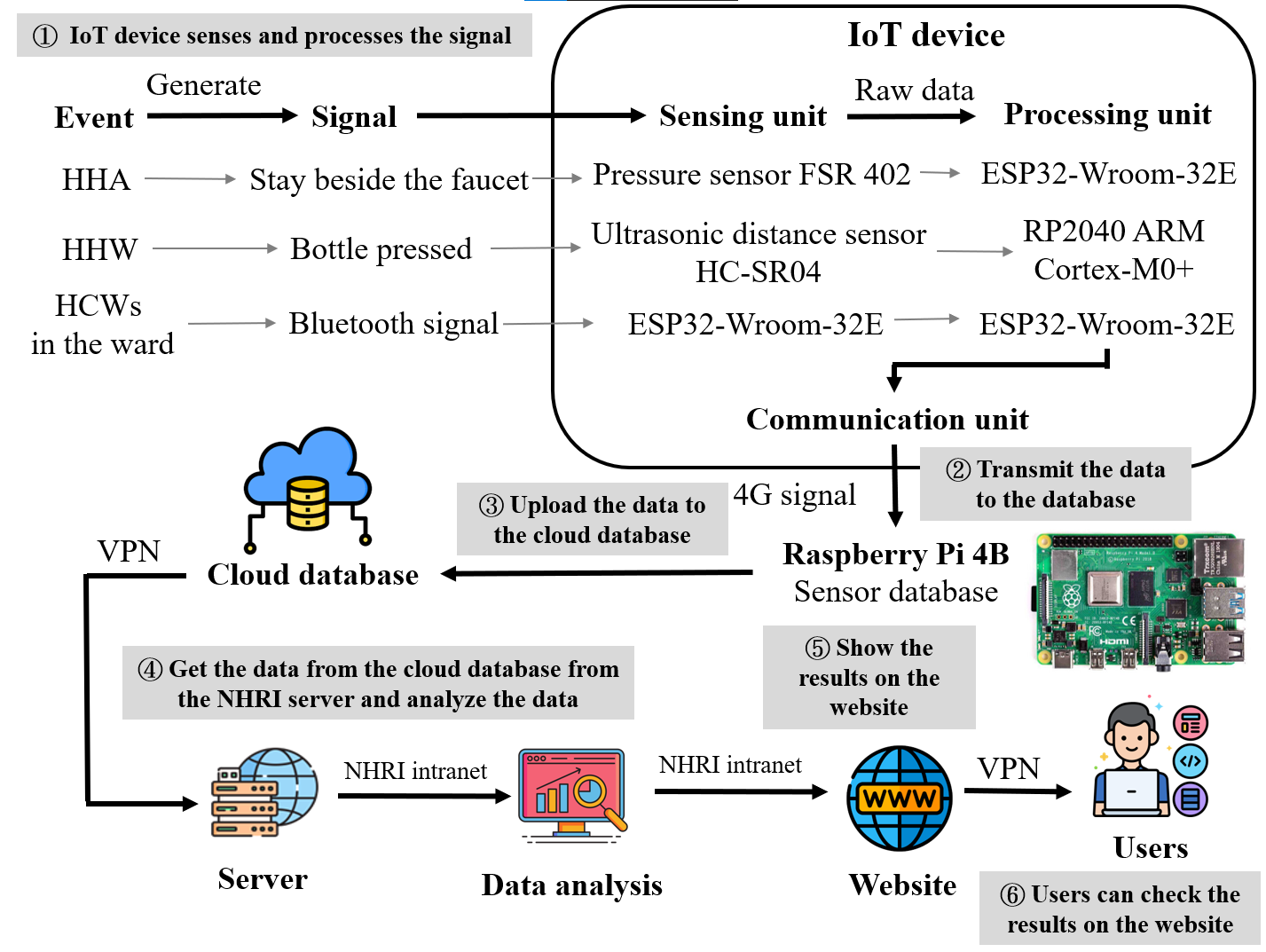


**Supplementary Figure 6.** The data flow of the cloud server is divided into six steps. In the first step, an IoT device senses and processes a signal. The data are subsequently transmitted to the database. The next step is to upload the data to the cloud database. The data can subsequently be obtained from the NHRI server and analyzed. Finally, users can check the detected hand hygiene status results on the website.


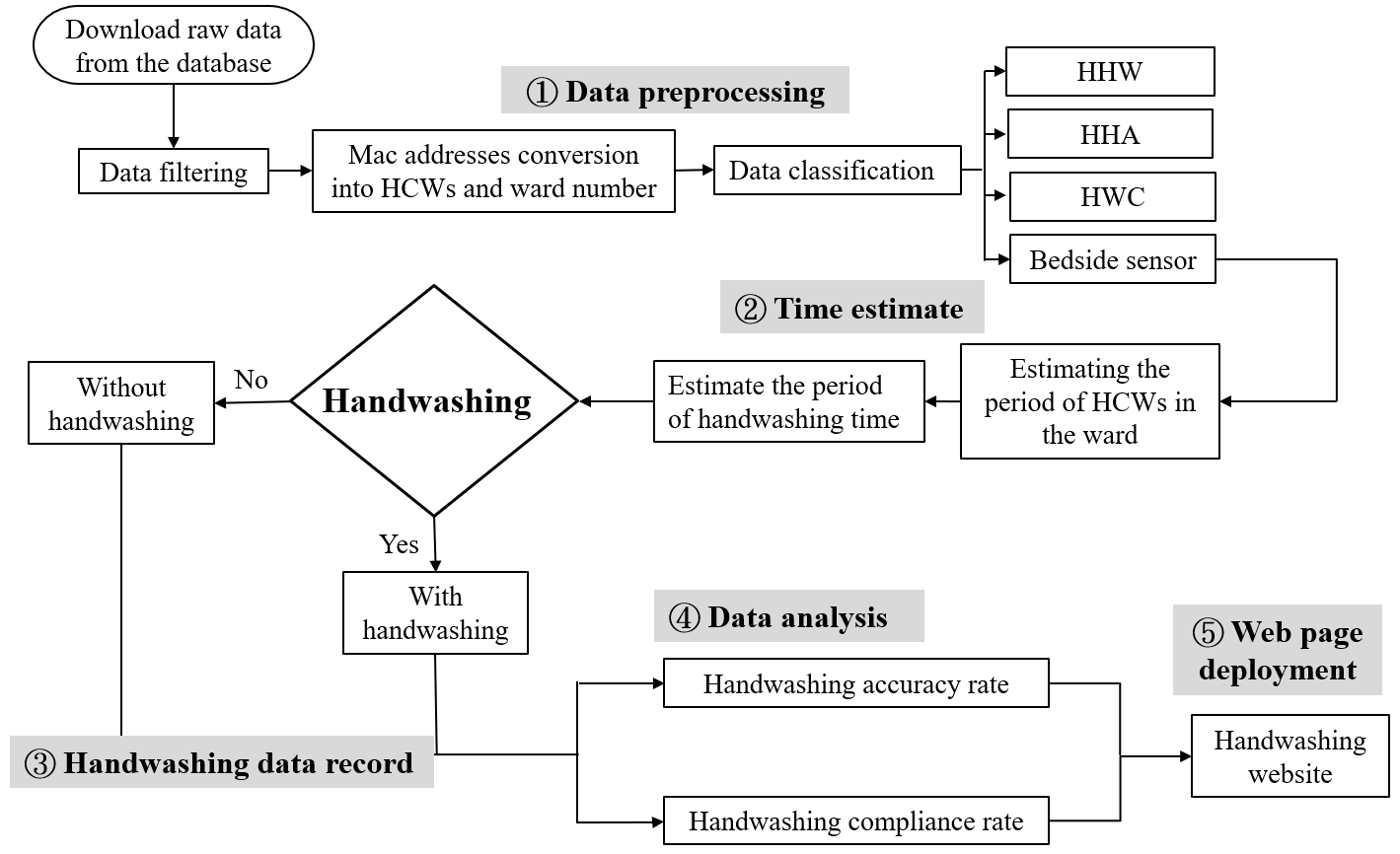


**Supplementary Figure 7.** The detailed clinical data compilation process is divided into five steps. The first step is data preprocessing, which includes data filtering and the conversion of MAC addresses into the identities of different detection devices. The second step was to estimate when each HCW was present in each ward and the duration of handwashing and then to record the handwashing information. The final steps involve analyzing and displaying the data on the handwashing detection system web page.

**References**

[1] W. H. Organization, "WHO guidelines on hand hygiene in health care," in *WHO guidelines on hand hygiene in health care*, 2009, pp. 270-270.

[2] *Cosmetic Hygiene and Safety Act,* M. o. H. a. Welfare, 2015.

[3] *Antibacterial cleaning product management,* M. o. H. a. Welfare, 2018.

[4] *Hand Hygiene Workbook,* M. o. H. a. W. Centers for Disease Control, 2012.
